# Supplementary material for: Defects in immune response to Toxoplasma gondii are associated with enhanced HIV-1-related neurocognitive impairment in co-infected patients
Source: PLoS One. 2023 May 24;18(5):e0285976. doi: 10.1371/journal.pone.0285976 (PMC10208516; doi:10.1371/journal.pone.0285976)
Supplement: S1 Table — (DOC) [file pone.0285976.s001.doc]

| **S1 Table - Description of Groups** | | | | | | |
| --- | --- | --- | --- | --- | --- | --- |
| **C1** |  |  |  |  |  |  |
| **Control 1** | **Sex** | **Age** | **CD4/ul** | **CD8/ul** | **Viral Load** | **Clinical Status** |
| **C1.1** | M | 41 | 618 | 704 | N/A | Asymptomatic |
| **C1.2** | F | 35 | 1,141 | 684 | N/A | Asymptomatic |
| **C1.3** | F | 38 | 1,021 | 221 | N/A | Asymptomatic |
| **C1.4** | F | 43 | 1,269 | 634 | N/A | Asymptomatic |
| **C1.5** | M | 32 | 798 | 399 | N/A | Asymptomatic |
| **C2** |  |  |  |  |  |  |
| **Control 2** | **Sex** | **Age** | **CD4/ul** | **CD8/ul** | **Viral Load** | **Clinical Status** |
| **C2.1** | M | 25 | 524 | 245 | N/A | Asymptomatic |
| **C2.2** | M | 34 | 384 | 576 | N/A | Asymptomatic |
| **C2.3** | M | 26 | 693 | 647 | N/A | Asymptomatic |
| **C2.4** | F | 17 | 505 | 232 | N/A | Asymptomatic |
| **C2.5** | F | 51 | 420 | 210 | N/A | Asymptomatic |
| **C2.6** | F | 44 | 696 | 553 | N/A | Asymptomatic |
| **C2.7** | M | 16 | 548 | 394 | N/A | Asymptomatic |
| **C2.8** | M | 31 | 933 | 270 | N/A | Asymptomatic |
| **C2.9** | M | 36 | 943 | 484 | N/A | Asymptomatic |
| **P1A** |  |  |  |  |  |  |
| **Patient 1A** | **Sex** | **Age** | **CD4/ul** | **CD8/ul** | **Viral Load** | **Clinical Status** |
| **P1A.1** | M | 23 | 512 | 1,139 | 11,568 | Asymptomatic |
| **P1A.2** | M | 38 | 627 | 1,499 | 51,286 | Asymptomatic |
| **P1A.3** | F | 23 | 645 | 1,431 | 185 | Asymptomatic |
| **P1A.4** | F | 24 | 441 | 1,063 | 907 | Asymptomatic |
| **P1A.5** | M | 29 | 490 | 595 | 1,216 | Adenopathies, weakness, depression |
| **P1A.6** | F | 31 | 400 | 714 | 28,611 | Asymptomatic |
| **P1A.7** | M | 43 | 792 | 984 | 27,810 | Asymptomatic |
| **P1A.8** | M | 23 | 412 | 938 | 46,770 | Asymptomatic |
| **P1A.9** | M | 29 | 390 | 1,478 | 22,214 | Kaposi's sarcoma |
| **P1A.10** | F | 36 | 945 | 648 | 774 | Asymptomatic |
| **P1A.11** | F | 47 | 676 | 892 | 4,482 | Asymptomatic |
| **P1B/C** |  |  |  |  |  |  |
| **Patient 1B/C** | **Sex** | **Age** | **CD4/ul** | **CD8/ul** | **Viral Load** | **Clinical Status** |
| **P1B/C.1** | M | 32 | 247 | 1,976 | 171,743 | Not Available |
| **P1B/C.2** | F | 37 | 280 | 893 | 19,688 | Not Available |
| **P1B/C.3** | M | 40 | 206 | 1,067 | 218,138 | Asymptomatic |
| **P1B/C.4** | M | 30 | 218 | 530 | 32,777 | Headache |
| **P1B/C.5** | M | 19 | 242 | 741 | 19,882 | Fever, weakness, diarrhea, vomiting |
| **P1B/C.6** | F | 34 | 261 | 363 | 3,465 | Flu |
| **P1B/C.7** | M | 24 | 331 | 539 | 19,818 | Asymptomatic |
| **P1B/C.8** | M | 45 | 218 | 545 | 6,885 | Asymptomatic |
| **P1B/C.9** | M | 45 | 205 | 497 | 29,099 | Dyspnea, thrombosis |
| **P1B/C.10** | M | 21 | 332 | 1,014 | 60,327 | Asymptomatic |
| **P1B/C.11** | F | 58 | 240 | 1,344 | 4,458 | Adenopathy |
| **P1B/C.12** | F | 35 | 251 | 426 | 46,604 | Asymptomatic |
| **P1B/C.13** | M | 37 | 275 | 454 | 117,622 | Weight loss |
| **P1B/C.14** | M | 30 | 218 | 1,185 | 6,750 | Fever, headache |
| **P1B/C.15** | M | 28 | 20 | 951 | 20,319 | Not Available |
| **P1B/C.16** | M | 50 | 75 | 705 | 192,322 | Pneumonia, thrombosis, oral candidiasis |
| **P1B/C.17** | M | 26 | 177 | 501 | 92,344 | TBC, HAD |
| **P1B/C.18** | M | 23 | 119 | 1,561 | >500,000 | Fever, cough |
| **P1B/C.19** | M | 31 | 170 | 1,134 | 8,875 | Fever, cough |
| **P1B/C.20** | M | 37 | 130 | 852 | 244,389 | Weight loss, respiratory disorder |
| **P1B/C.21** | M | 31 | 175 | 1,000 | >500,000 | TBC |
| **P1B/C.22** | M | 47 | 4 | 631 | 91,682 | Diarrhea, weight loss, consumption |
| **P1B/C.23** | M | 48 | 324 | 1,728 | 9,862 | Asymptomatic |
| **P1B/C.24** | M | 24 | 428 | 552 | 43,223 | Asymptomatic |
| **P1B/C.25** | F | 44 | 174 | 112 | Not Available | Diarrhea, weight loss |
| **P2A** |  |  |  |  |  |  |
| **Patient 2A** | **Sex** | **Age** | **CD4/ul** | **CD8/ul** | **Viral Load** | **Clinical Status** |
| **P2A.1** | M | 45 | 561 | 935 | 300 | Asymptomatic |
| **P2A.2** | M | 21 | 869 | 1,773 | 9,317 | Asymptomatic |
| **P2A.3** | F | 40 | 383 | 559 | 3,501 | Asymptomatic |
| **P2A.4** | M | 21 | 653 | 843 | 23,611 | Asymptomatic |
| **P2A.5** | F | 32 | 388 | 1,143 | 18,588 | Recurrent pneumonia |
| **P2A.6** | M | 20 | 1,409 | 1,445 | 14,535 | Weight loss, depression |
| **P2A.7** | M | 35 | 512 | 1,001 | 22,359 | Asymptomatic |
| **P2A.8** | M | 46 | 366 | 732 | 6,824 | Insomnia, headache |
| **P2A.9** | F | 41 | 403 | 806 | 4,381 | Asymptomatic |
| **P2B/C** |  |  |  |  |  |  |
| **Patient 2B/C** | **Sex** | **Age** | **CD4/ul** | **CD8/ul** | **Viral Load** | **Clinical Status** |
| **P2B/C.1** | M | 27 | 272 | 656 | 123,455 | Herpes |
| **P2B/C.2** | M | 38 | 345 | 954 | 110,489 | Fever, diarrhea, headache |
| **P2B/C.3** | F | 36 | 329 | 628 | 2,164 | Asymptomatic |
| **P2B/C.4** | F | 46 | 338 | 1,104 | 98,630 | Respiratory, cardiac and gastrointestinal disorders |
| **P2B/C.5** | F | 23 | 204 | 1,055 | 23,216 | Pneumonia |
| **P2B/C.6** | M | 30 | 244 | 2,123 | 20,453 | Asymptomatic |
| **P2B/C.7** | M | 53 | 309 | 1,487 | >500,000 | Fever, cough, weight loss |
| **P2B/C.8** | F | 44 | 331 | 677 | 1,022 | Asymptomatic |
| **P2B/C.9** | M | 28 | 259 | 346 | 78,526 | Fever |
| **P2B/C.10** | M | 33 | 82 | 575 | 159,069 | Not Available |
| **P2B/C.11** | M | 27 | 92 | 1,771 | 6,272 | Not Available |
| **P2B/C.12** | M | 43 | 14 | 835 | >500,000 | Temporary blindness |
| **P2B/C.13** | M | 50 | 98 | 442 | 29,731 | TBC |
| **P2B/C.14** | M | 36 | 49 | 1,066 | 35,011 | TBC |
| **P2B/C.15** | M | 36 | 107 | 447 | 72,287 | Pleomorphic adenoma, lymphadenopathies, fever |
| **P2B/C.16** | M | 42 | 23 | 797 | 67,670 | Diarrhea, weight loss |
| **P2B/C.17** | M | 62 | 53 | 600 | 22,674 | Not Available |
| **P2B/C.18** | M | 41 | 157 | 1,102 | 22,549 | Pneumonia |
| **P2B/C.19** | M | 30 | 312 | 737 | 16,595 | Asymptomatic |
| **P2B/C.20** | M | 41 | 263 | 1,336 | 17,128 | Facial paralysis |
| **P2B/C.21** | M | 33 | 216 | 1,269 | 10,405 | Asymptomatic |
| **P2B/C.22** | M | 48 | 56 | 644 | 28,776 | Pneumocystis pneumonia |
| **P2B/C.23** | M | 28 | 259 | 346 | 78,526 | Fever |
| **P2B/C.24** | M | 32 | 348 | 1,118 | 8,442 | Asymptomatic |

**Sex:** sex of the participant (M: male; F: female); **Age:** age of participants (years); **CD4/ul:** peripheral blood counts of CD4+ T lymphocytes (cells/μL); **CD8/ul:** peripheral blood counts of CD8+ T lymphocytes (cells/μL); **Viral Load:** HIV-1 viral load (copies of HIV-1 RNA/mL of plasma); **Clinical Status:** a brief description of patient’s signs and symptoms at the moment of his/her participation; **N/A:** Not applicable; **TBC:** pulmonary tuberculosis¸ **HAD:** HIV-1 associated dementia
