# Supplementary material for: Defects in immune response to Toxoplasma gondii are associated with enhanced HIV-1-related neurocognitive impairment in co-infected patients
Source: PLoS One. 2023 May 24;18(5):e0285976. doi: 10.1371/journal.pone.0285976 (PMC10208516; doi:10.1371/journal.pone.0285976)
Supplement: S2 Table — (DOC) [file pone.0285976.s002.doc]

| **S2 Table - Production of Cytokines** | | | | | |  |  |  |  |  |  |  |
| --- | --- | --- | --- | --- | --- | --- | --- | --- | --- | --- | --- | --- |
| **C1** | **IL-2** | | | **IL-10** | | | **TNF-α** | | | **IFN-γ** | | |
| **Control 1** | **Medium** | **PHA** | **STAg** | **Medium** | **PHA** | **SATg** | **Medium** | **PHA** | **SATg** | **Medium** | **PHA** | **SATg** |
| **C1.1** | **26** | **23** | **35** | **9** |  | **36** | **3** | **5** | **2** | **43** | **51** | **25** |
| **C1.2** | **17** | **32** | **18** |  | **354** |  | **7** |  | **12** | **23** | **10821** | **43** |
| **C1.3** | **16** | **23** | **17** | **3** | **486** | **3** | **2** | **8** | **3** | **29** | **48** | **36** |
| **C1.4** | **17** | **18** | **19** | **9** | **750** | **9** | **3** | **8** | **2** | **27** | **41** | **36** |
| **C1.5** | **16** | **15** | **59** | **6** | **906** | **16** | **4** | **108** | **20** | **18** | **9047** |  |
| **C2** | **IL-2** | | | **IL-10** | | | **TNF-α** | | | **IFN-γ** | | |
| **Control 2** | **Medium** | **PHA** | **SATg** | **Medium** | **PHA** | **SATg** | **Medium** | **PHA** | **SATg** | **Medium** | **PHA** | **SATg** |
| **C2.1** | **14** | **11** | **196** | **4** | **1661** | **60** | **2** | **214** | **422** | **28** | **37819^a^** | **41280^a^** |
| **C2.2** | **13** | **11** | **143** | **3** | **750** | **32** | **3** | **49** | **82** | **38** | **8074** | **16263** |
| **C2.3** | **18** | **17** | **38** | **10** | **498** | **28** | **3** | **68** | **50** | **39** | **1483** | **3242** |
| **C2.4** | **19** | **19** | **188** | **3** | **1185** | **35** | **3** | **61** | **460** | **32** | **2391** | **37520^a^** |
| **C2.5** | **22** | **16** | **98** | **10** | **800** | **49** | **6** | **473** | **54** |  |  |  |
| **C2.6** | **18** | **16** | **57** | **25** | **1064** | **40** | **4** | **75** |  | **23** | **326** |  |
| **C2.7** | **17** | **26** | **32** |  | **914** | **101** | **17** |  |  | **92** | **74225^a^** | **37918^a^** |
| **C2.8** | **18** | **22** | **24** |  |  |  |  | **527** | **286** |  |  |  |
| **C2.9** | **25** | **20** | **20** |  |  |  |  |  |  |  |  |  |
| **P1A** | **IL-2** | | | **IL-10** | | | **TNF-α** | | | **IFN-γ** | | |
| **Patient 1A** | **Medium** | **PHA** | **SATg** | **Medium** | **PHA** | **SATg** | **Medium** | **PHA** | **SATg** | **Medium** | **PHA** | **SATg** |
| **P1A.2** | **18** | **28** | **16** | **18** | **208** | **35** | **3** | **757** | **3** | **31** | **5556** | **33** |
| **P1A.6** | **14** | **18** | **15** |  |  |  |  |  |  |  |  |  |
| **P1A.7** | **19** | **15** | **19** | **3** | **2384** | **63** | **3** | **831** | **4** | **31** | **34044^a^** | **37** |
| **P1A.10** | **20** | **17** | **20** | **13** | **1762** | **73** | **3** | **1100** | **7** | **39** | **34776^a^** | **54** |
| **P1A.11** | **18** | **18** | **20** | **4** | **631** | **43** | **2** | **1441** | **21** | **33** | **25029^a^** | **46** |
| **P1B/C** | **IL-2** | | | **IL-10** | | | **TNF-α** | | | **IFN-γ** | | |
| **Patient 1B/C** | **Medium** | **PHA** | **SATg** | **Medium** | **PHA** | **SATg** | **Medium** | **PHA** | **SATg** | **Medium** | **PHA** | **SATg** |
| **P1B/C.9** | **18** | **18** | **17** | **19** | **2514** | **162** | **3** | **767** | **6** | **29** | **20010^a^** | **42** |
| **P1B/C.10** | **19** | **19** | **17** | **105** | **1244** | **127** | **4** | **2310** | **4** | **33** | **22243^a^** | **35** |
| **P1B/C.12** | **22** | **23** | **18** | **137** | **1984** | **20** | **36** | **1687** | **8** | **58** | **54711^a^** | **45** |
| **P1B/C.13** | **17** | **23** | **16** | **6** | **344** | **7** | **5** | **411** | **7** | **33** | **10341** | **38** |
| **P1B/C.16** | **18** | **20** | **18** | **249** | **594** | **379** | **13** | **1134** | **33** | **35** | **7440** | **23** |
| **P1B/C.19** | **20** | **22** | **21** | **32** | **1618** | **167** | **5** | **316** | **7** | **56** | **769** | **48** |
| **P1B/C.24** | **16** | **19** | **15** |  |  |  |  |  |  |  |  |  |
| **P1B/C.25** | **17** | **17** |  | **75** | **623** |  | **12** | **334** |  | **39** |  |  |
| **P2A** | **IL-2** | | | **IL-10** | | | **TNF-α** | | | **IFN-γ** | | |
| **Patient 2A** | **Medium** | **PHA** | **SATg** | **Medium** | **PHA** | **SATg** | **Medium** | **PHA** | **SATg** | **Medium** | **PHA** | **SATg** |
| **P2A.1** | **12** | **13** | **12** | **6** | **2787** | **14** | **7** | **943** | **4** | **41** | **8666** | **43** |
| **P2A.3** | **13** | **16** | **21** |  |  |  |  |  |  |  |  |  |
| **P2A.4** | **21** | **21** | **24** | **5** | **780** | **10** | **8** | **580** | **62** |  | **50854^a^** |  |
| **P2A.5** | **22** | **21** | **21** | **5** | **1051** | **8** | **5** | **1274** | **7** | **43** | **3766** | **68** |
| **P2A.6** | **15** | **25** | **20** | **24** | **791** | **40** | **8** | **1163** | **18** | **37** | **71385^a^** | **277** |
| **P2A.7** | **11** | **68** | **18** | **6** | **3428** | **185** | **2** | **3123** | **6** | **30** | **31280^a^** | **42** |
| **P2A.8** | **21** | **18** | **23** | **17** | **187** | **17** |  |  |  | **58** | **27951^a^** | **1964** |
| **P2A.9** | **17** | **22** | **21** | **34** | **182** | **142** | **20** | **884** | **135** | **18** | **11292** | **107** |
| **P2B/C** | **IL-2** | | | **IL-10** | | | **TNF-α** | | | **IFN-γ** | | |
| **Patient 2B/C** | **Medium** | **PHA** | **SATg** | **Medium** | **PHA** | **SATg** | **Medium** | **PHA** | **SATg** | **Medium** | **PHA** | **SATg** |
| **P2B/C.1** | **12** | **12** | **12** |  |  |  | **8** | **1929** | **80** | **39** | **33603^a^** | **102** |
| **P2B/C.5** | **20** | **19** | **20** | **3** | **1478** | **42** | **3** | **2364** | **10** | **31** | **35101^a^** | **45** |
| **P2B/C.6** | **16** | **20** | **15** | **46** | **180** | **68** | **42** | **1335** | **94** | **36** | **20398^a^** | **199** |
| **P2B/C.7** | **14** | **12** | **15** | **27** | **227** | **68** |  |  |  | **43** | **31413^a^** | **65** |
| **P2B/C.8** | **19** | **15** | **27** | **5** | **507** | **11** | **4** | **203** | **206** | **24** | **32945^a^** |  |
| **P2B/C.12** | **18** | **18** | **21** | **9** | **824** | **17** | **2** | **52** | **4** | **37** |  | **39** |
| **P2B/C.13** | **17** | **19** | **18** | **11** | **473** | **129** | **6** | **874** | **76** | **40** | **12347** | **53** |
| **P2B/C.15** | **15** | **12** | **13** | **49** | **427** | **32** | **31** | **469** | **6** | **69** | **1436** | **21** |
| **P2B/C.18** | **17** | **13** | **17** | **38** | **668** | **171** | **10** | **3130** | **146** | **44** | **37327^a^** | **1089** |
| **P2B/C.19** | **19** | **37** | **23** | **28** | **459** | **84** |  |  |  |  |  |  |
| **P2B/C.20** | **18** | **21** | **18** | **39** | **1030** | **235** | **3** | **531** | **8** | **35** | **12381** | **61** |
| **P2B/C.21** | **19** | **20** | **24** | **16** | **231** | **22** | **5** | **1809** | **66** | **31** | **5201** | **887** |
| **P2B/C.22** | **18** | **19** | **21** | **16** | **1560** | **334** | **3** | **859** | **234** | **24** | **10802** | **774** |

PBMC of the different groups of participants were cultured for 72 hours in basal/non-stimulated conditions (Medium), in the presence of the polyclonal activator phytohemagglutinin (PHA, 5 μg/mL) or in the presence of soluble antigens of tachyzoites of *T. gondii* (SATg, 1 μg/mL). IL-2, IL-10, TNF-α and IFN-γ were determined in the supernatants of cultures and are expressed in pg/mL.

**^a^** Values of IFN-γ greater than 20000 pg/mL are considered beyond the range of the calibration curve.
