# Supplementary material for: Defects in immune response to Toxoplasma gondii are associated with enhanced HIV-1-related neurocognitive impairment in co-infected patients
Source: PLoS One. 2023 May 24;18(5):e0285976. doi: 10.1371/journal.pone.0285976 (PMC10208516; doi:10.1371/journal.pone.0285976)
Supplement: S3 Table — (DOC) [file pone.0285976.s003.doc]

**S3 Table – Visual P300 Latency**

| **P1A** | **Visual P300 - Latency** | | | | | | |  |  |  |  |  |  |  |  |  |  |  |  |  |
| --- | --- | --- | --- | --- | --- | --- | --- | --- | --- | --- | --- | --- | --- | --- | --- | --- | --- | --- | --- | --- |
| **Patient 1A** | **Fp1** | **Fp2** | **F3** | **F4** | **C3** | **C4** | **P3** | **P4** | **O1** | **O2** | **F7** | **F8** | **T3** | **T4** | **T5** | **T6** | **Fz** | **Cz** | **Pz** | **Oz** |
| **P1A.1** | 411 | 407 | 411 | 415 | 411 | 411 | 407 | 403 | 392 | 388 | 407 | 411 | 415 | 411 | 403 | 403 | 415 | 415 | 403 | 392 |
| **P1A.2** | 442 | 454 | 442 | 446 | 442 | 439 | 446 | 435 | 450 | 442 | 454 | 446 | 446 | 442 | 454 | 446 | 446 | 442 | 439 | 454 |
| **P1A.3** | 403 | 407 | 392 | 400 | 388 | 396 | 380 | 384 | 362 | 380 | 388 | 405 | 384 | 400 | 376 | 392 | 400 | 392 | 384 | 376 |
| **P1A.4** | 474 | 482 | 419 | 431 | 392 | 418 | 384 | 396 | 368 | 384 | 346 | 335 | 394 | 411 | 380 | 496 | 435 | 454 | 396 | 388 |
| **P1A.5** | 536 | 521 | 454 | 435 | 446 | 431 | 419 | 411 | 400 | 403 | 478 | 485 | 474 | 458 | 462 | 423 | 439 | 435 | 400 | 403 |
| **P1A.6** | 360 | 357 | 372 | 380 | 372 | 384 | 368 | 380 | 364 | 400 | 360 | 380 | 380 | 392 | 376 |  | 376 | 376 | 372 | 364 |
| **P1A.7** | 364 | 376 | 368 | 388 | 368 | 384 | 360 | 368 | 349 | 368 | 368 | 380 | 360 | 388 | 353 | 392 | 380 | 380 | 357 | 353 |
| **P1A.8** | 384 | 396 | 380 | 368 | 376 | 368 | 388 | 364 | 403 | 392 | 372 | 360 | 380 | 364 | 380 | 376 | 376 | 376 | 384 | 396 |
| **P1A.9** |  |  | 376 | 400 | 396 | 392 | 392 | 388 | 392 | 388 | 337 | 392 | 396 | 396 | 400 | 396 | 403 | 388 | 388 | 392 |
| **P1B/C** | **Visual P300 - Latency** | | | | | | |  |  |  |  |  |  |  |  |  |  |  |  |  |
| **Patient 1B/C** | **Fp1** | **Fp2** | **F3** | **F4** | **C3** | **C4** | **P3** | **P4** | **O1** | **O2** | **F7** | **F8** | **T3** | **T4** | **T5** | **T6** | **Fz** | **Cz** | **Pz** | **Oz** |
| **P1B/C.1** | 360 | 364 | 364 | 368 | 357 | 360 | 357 | 357 | 353 | 357 | 364 | 364 | 360 | 360 | 353 | 357 | 364 | 357 | 353 | 353 |
| **P1B/C.2** | 400 | 423 | 392 | 400 | 388 | 396 | 380 | 392 | 376 | 384 | 392 | 392 | 388 | 396 | 384 | 396 | 396 | 392 | 388 | 384 |
| **P1B/C.3** | 372 | 368 | 360 | 380 | 376 | 380 | 376 | 376 | 372 | 372 | 376 | 380 | 376 | 380 | 372 | 380 | 384 | 380 | 380 | 372 |
| **P1B/C.4** | 388 | 388 | 396 | 396 | 392 | 396 | 388 | 388 | 384 | 376 | 396 | 392 | 407 | 407 | 392 | 396 | 396 | 396 | 388 | 380 |
| **P1B/C.5** | 360 | 357 | 368 | 372 | 372 | 396 | 396 | 400 | 342 | 400 | 364 | 380 | 372 | 346 | 596 | 403 | 368 | 372 | 392 | 400 |
| **P1B/C.6** | 489 | 505 | 482 | 485 | 431 | 478 | 482 | 474 | 403 | 435 | 560 | 485 | 497 | 485 | 435 | 478 | 482 | 478 | 482 | 407 |
| **P1B/C.7** | 411 | 400 | 411 | 411 | 329 | 415 | 392 | 400 | 388 | 396 | 411 | 411 | 396 | 411 | 392 | 298 | 321 | 329 | 329 | 392 |
| **P1B/C.8** | 360 | 357 | 349 | 349 | 329 | 349 | 337 | 341 | 317 | 337 | 353 | 353 | 345 | 349 | 337 | 345 | 341 | 349 | 341 | 333 |
| **P1B/C.9** | 415 | 388 | 442 | 435 | 462 | 442 | 427 | 439 | 423 | 415 | 419 | 423 | 435 | 439 | 446 | 439 | 431 | 435 | 431 | 423 |
| **P1B/C.10** | 392 | 392 | 383 | 376 | 376 | 380 | 407 | 396 | 357 | 349 | 388 | 380 | 396 | 392 | 403 | 380 | 380 | 372 | 441 | 349 |
| **P1B/C.11** | 423 | 431 | 427 | 439 | 431 | 431 | 423 | 415 | 423 | 415 | 384 | 454 | 427 | 372 | 423 | 415 | 442 | 431 | 427 | 414 |
| **P1B/C.12** | 428 | 427 | 403 | 384 | 400 | 388 | 407 | 392 | 400 | 400 | 400 | 392 | 400 | 392 | 400 | 400 | 396 | 384 | 396 | 396 |
| **P1B/C.13** | 372 | 337 | 376 | 368 | 368 | 360 | 364 | 360 | 360 | 360 | 372 | 353 | 372 | 357 | 364 |  | 372 | 364 | 360 | 360 |
| **P1B/C.14** | 380 | 388 | 396 | 396 | 403 | 400 | 407 | 407 | 439 | 407 | 396 | 388 | 403 | 403 | 450 | 411 | 396 | 400 | 407 | 427 |
| **P1B/C.15** | 407 | 407 | 392 | 392 | 411 | 403 | 396 | 403 | 384 | 392 | 400 | 400 | 403 | 400 | 396 | 403 | 400 | 407 | 396 | 376 |
| **P1B/C.16** | 560 | 564 | 532 | 540 | 517 | 564 | 493 | 474 | 446 | 446 | 528 | 544 | 521 | 489 | 470 | 485 | 536 | 517 | 485 | 446 |
| **P1B/C.17** | 400 | 392 | 403 | 403 | 403 | 407 | 400 | 396 | 396 | 396 | 400 | 392 | 400 | 392 | 403 | 400 | 407 | 411 | 400 | 396 |
| **P1B/C.18** | 314 | 314 | 329 | 317 | 345 | 329 | 341 | 337 | 368 | 341 | 333 | 353 | 357 | 353 | 364 | 353 | 321 | 329 | 341 | 345 |
| **P1B/C.19** |  | 329 | 407 | 396 | 372 | 372 | 357 | 372 | 360 | 380 |  | 400 | 345 | 376 | 353 | 368 | 396 | 368 | 368 | 368 |
| **P1B/C.20** | 450 | 474 | 446 | 446 | 450 | 474 | 403 | 384 | 396 | 388 | 472 | 431 | 442 | 442 | 415 | 388 | 454 | 470 | 392 | 392 |
| **P1B/C.21** | 411 | 384 | 411 | 388 | 400 | 396 | 400 | 388 | 400 | 396 | 419 | 388 | 403 | 388 | 396 | 384 | 411 | 407 | 400 | 396 |
| **P1B/C.22** | 427 | 403 | 427 | 419 | 423 | 415 | 423 | 419 | 431 | 415 | 466 | 396 | 427 | 407 | 431 | 411 | 431 | 419 | 419 | 419 |
| **P1B/C.23** | 329 | 364 | 376 | 376 | 380 | 380 | 368 | 376 | 364 | 341 | 337 | 407 | 372 | 380 | 364 | 376 | 380 | 376 | 427 | 388 |
| **P2A** | **Visual P300 - Latency** | | | | | | |  |  |  |  |  |  |  |  |  |  |  |  |  |
| **Patient 2A** | **Fp1** | **Fp2** | **F3** | **F4** | **C3** | **C4** | **P3** | **P4** | **O1** | **O2** | **F7** | **F8** | **T3** | **T4** | **T5** | **T6** | **Fz** | **Cz** | **Pz** | **Oz** |
| **P2A.1** | 446 | 454 | 450 | 450 | 450 | 442 | 446 | 442 | 442 | 439 | 442 | 450 | 454 | 439 | 450 | 435 | 450 | 450 | 446 | 439 |
| **P2A.2** | 364 | 345 | 337 | 341 | 333 | 337 | 333 | 333 | 337 | 337 | 345 | 345 | 333 | 337 | 337 | 341 | 341 | 337 | 333 | 337 |
| **P2A.3** | 360 | 349 | 364 | 360 | 353 | 353 | 353 | 357 | 353 | 353 | 353 | 357 | 353 | 360 | 360 | 357 | 360 | 360 | 349 | 353 |
| **P2A.4** | 329 | 360 | 388 | 384 | 380 | 380 | 376 | 376 | 376 | 372 | 380 | 376 | 380 | 380 | 380 | 380 | 388 | 380 | 376 | 372 |
| **P2B/C** | **Visual P300 - Latency** | | | | | | |  |  |  |  |  |  |  |  |  |  |  |  |  |
| **Patient 2B/C** | **Fp1** | **Fp2** | **F3** | **F4** | **C3** | **C4** | **P3** | **P4** | **O1** | **O2** | **F7** | **F8** | **T3** | **T4** | **T5** | **T6** | **Fz** | **Cz** | **Pz** | **Oz** |
| **P2B/C.1** | 400 | 415 | 419 | 431 | 419 | 431 | 419 | 435 | 415 | 427 | 423 | 423 | 419 | 466 | 419 | 454 | 427 | 423 | 419 | 419 |
| **P2B/C.2** | 442 | 446 | 384 | 388 | 380 | 380 | 380 | 376 | 376 | 372 | 396 | 392 | 388 | 384 | 384 | 380 | 388 | 384 | 380 | 372 |
| **P2B/C.3** | 314 | 329 | 360 | 364 | 368 | 368 | 368 | 368 | 360 | 368 | 353 | 357 | 364 | 368 | 364 | 372 | 368 | 372 | 368 | 364 |
| **P2B/C.4** | 466 | 482 | 454 | 454 | 466 | 470 | 478 | 485 | 321 | 485 | 454 | 478 | 380 | 482 | 317 | 478 | 458 | 466 | 478 | 481 |
| **P2B/C.5** | 540 | 540 | 540 | 540 | 525 | 540 | 530 | 521 | 509 | 528 | 536 | 544 | 521 | 536 | 513 | 540 | 540 | 525 | 513 | 489 |
| **P2B/C.6** | 376 | 353 | 392 | 368 | 357 | 360 | 364 | 364 | 368 | 368 | 380 | 349 | 368 | 372 | 368 | 372 | 396 | 364 | 364 | 360 |
| **P2B/C.7** | 403 | 392 | 400 | 400 | 407 | 396 | 411 | 400 | 431 | 431 | 400 | 396 | 407 | 392 | 423 | 431 | 407 | 407 | 407 | 435 |
| **P2B/C.8** | 325 | 425 | 392 | 372 | 427 | 427 | 439 | 431 | 407 | 439 | 360 | 345 | 403 | 415 | 419 | 435 | 415 | 431 | 442 | 427 |
| **P2B/C.9** | 341 | 353 | 353 | 353 | 345 | 354 | 353 | 349 | 353 | 349 | 345 | 349 | 353 | 353 | 357 | 353 | 357 | 357 | 353 | 349 |
| **P2B/C.10** |  |  | 431 | 439 | 439 | 439 | 435 | 435 | 411 | 431 | 427 | 427 | 423 | 431 | 419 | 431 | 439 | 528 | 435 | 415 |
| **P2B/C.11** | 360 | 353 | 372 | 372 | 388 | 388 | 392 | 400 | 346 | 400 | 370 | 368 | 400 | 396 | 396 | 403 | 372 | 388 | 396 | 396 |
| **P2B/C.12** | 474 | 482 | 458 | 446 | 442 | 446 | 435 | 439 | 435 | 435 | 454 | 446 | 439 | 435 | 435 | 435 | 458 | 446 | 439 | 435 |
| **P2B/C.13** | 388 | 388 | 407 | 407 | 411 | 415 | 411 | 411 | 345 | 403 | 388 | 407 | 407 | 415 | 345 | 415 | 411 | 411 | 407 | 407 |
| **P2B/C.14** | 454 | 458 | 439 | 439 | 439 | 442 | 431 | 439 | 372 | 427 | 446 | 439 | 364 | 442 | 384 | 435 | 442 | 442 | 435 | 431 |
| **P2B/C.15** | 439 | 435 | 400 | 407 | 400 | 411 | 407 | 415 | 403 | 411 | 392 | 423 | 396 | 431 | 411 | 407 | 407 | 403 | 407 | 407 |
| **P2B/C.16** | 466 | 446 | 380 | 376 | 376 | 376 | 368 | 372 | 364 | 364 | 372 | 364 | 392 | 372 | 364 | 364 | 388 | 388 | 380 | 372 |
| **P2B/C.17** | 642 | 579 | 575 | 560 | 462 | 478 | 454 | 470 | 462 | 470 |  | 536 | 458 | 505 | 458 | 462 | 579 | 470 | 462 | 470 |
| **P2B/C.18** | 501 | 501 | 427 | 419 | 419 | 415 | 435 | 435 | 400 | 419 | 446 | 446 | 439 | 431 | 427 | 439 | 431 | 423 | 435 | 427 |
| **P2B/C.19** | 384 | 384 | 384 | 384 | 372 | 372 | 376 | 376 | 368 | 376 | 380 | 372 | 380 | 372 | 372 | 364 | 384 | 376 | 376 | 376 |
| **Control** | **Visual P300 - Latency** | | | | | | |  |  |  |  |  |  |  |  |  |  |  |  |  |
| VIH(-) | **Fp1** | **Fp2** | **F3** | **F4** | **C3** | **C4** | **P3** | **P4** | **O1** | **O2** | **F7** | **F8** | **T3** | **T4** | **T5** | **T6** | **Fz** | **Cz** | **Pz** | **Oz** |
| CNeu.1 | 341 | 376 | 333 | 384 | 407 | 415 | 392 | 439 | 376 | 403 | 333 | 337 | 380 | 489 | 384 | 317 | 396 | 419 | 415 | 380 |
| CNeu.2 | 403 | 403 | 419 | 411 | 396 | 392 | 376 | 372 | 345 | 357 | 435 | 419 | 427 | 423 | 423 | 423 | 415 | 407 | 368 | 370 |
| CNeu.3 | 376 | 368 | 384 | 388 | 388 | 384 | 396 | 392 | 388 | 400 | 384 | 384 | 396 | 415 | 411 | 411 | 380 | 380 | 384 | 370 |
| CNeu.4 | 368 | 368 | 368 | 353 | 400 | 372 | 403 | 384 | 388 | 380 | 372 | 372 | 419 | 380 | 400 | 380 | 349 | 376 | 384 | 374 |
| CNeu.5 | 380 | 431 | 384 | 392 | 384 | 384 | 376 | 372 | 360 | 357 | 439 | 430 | 403 | 384 | 388 | 372 | 388 | 380 | 364 | 370 |
| CNeu.6 | 442 | 427 | 411 | 411 | 419 | 403 | 400 | 392 | 368 | 372 | 454 | 435 | 419 | 427 | 396 | 400 | 419 | 415 | 400 | 370 |
| CNeu.7 | 376 | 349 | 388 | 368 | 392 | 384 | 384 | 380 | 372 | 372 | 384 | 368 | 388 | 376 | 380 | 376 | 380 | 392 | 376 | 376 |
| CNeu.8 | 392 | 396 | 400 | 407 | 396 | 407 | 403 | 403 | 388 | 380 | 407 | 427 | 415 | 439 | 431 | 419 | 388 | 384 | 388 | 370 |
| CNeu.9 | 333 | 329 | 388 | 384 | 392 | 392 | 388 | 388 | 368 | 364 | 415 | 396 | 403 | 396 | 388 | 368 | 380 | 380 | 380 | 375 |
| CNeu.10 | 419 | 407 | 403 | 392 | 388 | 384 | 372 | 372 | 360 | 357 | 407 | 411 | 388 | 384 | 368 | 321 | 400 | 384 | 364 | 368 |
| CNeu.11 | 431 | 431 | 407 | 407 | 407 | 411 | 403 | 411 | 278 | 294 | 415 | 400 | 392 | 396 | 329 | 353 | 415 | 411 | 396 | 350 |
| CNeu.12 | 396 | 403 | 392 | 403 | 400 | 400 | 403 | 396 | 396 | 392 | 403 | 376 | 427 | 388 | 407 | 392 | 403 | 400 | 392 | 388 |
| CNeu.13 | 372 | 364 | 376 | 376 | 380 | 376 | 372 | 372 | 364 | 368 | 380 | 372 | 376 | 372 | 372 | 368 | 380 | 380 | 372 | 364 |
| CNeu.14 | 357 | 357 | 396 | 388 | 407 | 396 | 380 | 388 | 376 | 384 | 407 | 400 | 396 | 388 | 360 | 364 | 396 | 392 | 392 | 396 |
| CNeu.15 | 263 | 263 | 333 | 333 | 341 | 345 | 345 | 345 | 345 | 345 | 337 | 335 | 349 | 357 | 357 | 341 | 333 | 337 | 345 | 357 |
| CNeu.16 | 357 | 360 | 388 | 384 | 388 | 388 | 384 | 392 | 368 | 384 | 376 | 372 | 384 | 400 | 380 | 384 | 392 | 388 | 384 | 384 |
| CNeu.17 | 357 | 357 | 384 | 380 | 392 | 392 | 403 | 396 | 396 | 392 | 317 | 317 | 392 | 388 | 400 | 388 | 384 | 396 | 400 | 396 |
| CNeu.18 | 294 | 282 | 403 | 403 | 400 | 326 | 403 | 396 | 376 | 380 | 415 | 403 | 415 | 400 | 384 | 396 | 407 | 407 | 392 | 376 |
| CNeu.19 | 372 | 322 | 364 | 376 | 372 | 368 | 372 | 368 | 360 | 368 | 360 | 372 | 376 | 374 | 364 | 372 | 372 | 372 | 380 | 380 |
| CNeu.20 | 250 | 353 | 384 | 380 | 364 | 380 | 372 | 372 | 368 | 368 | 358 | 372 | 345 | 376 | 380 | 372 | 380 | 376 | 368 | 368 |
| CNeu.21 | 360 | 376 | 372 | 376 | 376 | 376 | 392 | 384 | 400 | 400 | 368 | 372 | 380 | 392 | 400 | 364 | 368 | 372 | 380 | 396 |
| CNeu.22 | 275 | 282 | 310 | 310 | 317 | 317 | 321 | 314 | 321 | 306 | 298 | 310 | 321 | 314 | 329 | 306 | 317 | 321 | 321 | 317 |
| CNeu.23 | 316 | 372 | 388 | 380 | 392 | 392 | 396 | 396 | 396 | 392 | 384 | 380 | 396 | 392 | 403 | 388 | 384 | 392 | 396 | 400 |
| CNeu.24 | 391 | 392 | 396 | 396 | 388 | 392 | 384 | 384 | 384 | 380 | 392 | 364 | 392 | 384 | 392 | 384 | 403 | 322 | 380 | 376 |
| CNeu.25 | 300 | 372 | 368 | 368 | 364 | 368 | 360 | 364 | 353 | 357 | 368 | 368 | 360 | 368 | 360 | 368 | 368 | 368 | 360 | 349 |

Mean of latency values at each electrode location (according to the 10/20 International System [40]) are expressed in milliseconds (ms)
