# Supplementary material for: Defects in immune response to Toxoplasma gondii are associated with enhanced HIV-1-related neurocognitive impairment in co-infected patients
Source: PLoS One. 2023 May 24;18(5):e0285976. doi: 10.1371/journal.pone.0285976 (PMC10208516; doi:10.1371/journal.pone.0285976)
Supplement: S4 Table — (DOC) [file pone.0285976.s004.doc]

**S4 Table – Auditory P300 Latency**

| **P1A** | **Auditory P300 - Latency** | | | | | | | | |  |  |  |  |  |  |  |  |  |  |  |
| --- | --- | --- | --- | --- | --- | --- | --- | --- | --- | --- | --- | --- | --- | --- | --- | --- | --- | --- | --- | --- |
| **Patient 1A** | **Fp1** | **Fp2** | **F3** | **F4** | **C3** | **C4** | **P3** | **P4** | **O1** | **O2** | **F7** | **F8** | **T3** | **T4** | **T5** | **T6** | **Fz** | **Cz** | **Pz** | **Oz** |
| **P1A.1** | 415 | 423 | 333 | 349 | 345 | 349 | 353 | 353 | 353 | 353 | 349 | 345 | 337 | 349 | 337 | 349 | 341 | 349 | 353 | 353 |
| **P1A.2** | 310 | 314 | 317 | 329 | 321 | 333 | 329 | 333 | 360 | 376 | 306 | 314 | 314 | 325 | 325 | 337 | 329 | 329 | 329 | 360 |
| **P1A.3** | 415 | 392 | 364 | 376 | 329 | 337 | 321 | 325 | 310 | 325 | 364 | 376 | 337 | 345 | 314 | 333 | 372 | 325 | 321 | 317 |
| **P1A.4** | 489 | 493 | 485 | 513 | 509 | 528 | 517 | 521 | 525 | 528 | 478 | 536 | 505 | 544 | 517 | 532 | 505 | 509 | 591 | 517 |
| **P1A.5** | 384 | 396 | 384 | 392 | 376 | 384 | 368 | 372 | 353 | 372 | 396 | 392 | 384 | 388 | 364 | 380 | 388 | 372 | 364 | 357 |
| **P1A.6** | 431 |  | 442 | 454 | 501 | 450 | 427 | 431 | 427 | 435 |  | 462 | 462 | 462 | 415 | 446 | 446 | 466 | 439 | 431 |
| **P1A.7** | 333 | 333 | 337 | 349 | 337 | 345 | 337 | 345 | 329 | 392 | 333 | 349 | 329 | 353 | 329 | 364 | 341 | 341 | 337 | 341 |
| **P1A.8** | 446 | 442 | 431 | 431 | 427 | 427 | 427 | 403 | 427 | 427 | 439 | 446 | 442 |  | 431 | 431 | 376 | 423 | 423 | 427 |
| **P1A.9** | 345 | 341 | 341 | 337 | 329 | 329 | 325 | 321 | 314 | 321 | 341 | 337 | 341 | 325 | 329 | 325 | 337 | 325 | 321 | 325 |
| **P1B/C** | **Auditory P300 - Latency** | | | | | | | | |  |  |  |  |  |  |  |  |  |  |  |
| **Patient 1B/C** | **Fp1** | **Fp2** | **F3** | **F4** | **C3** | **C4** | **P3** | **P4** | **O1** | **O2** | **F7** | **F8** | **T3** | **T4** | **T5** | **T6** | **Fz** | **Cz** | **Pz** | **Oz** |
| **P1B/C.1** | 364 | 364 | 360 | 364 | 364 | 360 | 360 | 357 | 357 | 349 | 360 | 360 | 360 | 349 | 360 | 353 | 364 | 368 | 360 | 353 |
| **P1B/C.2** | 427 | 407 | 376 | 368 | 368 | 345 | 392 | 337 | 380 | 360 | 372 | 360 | 372 | 345 | 372 | 349 | 360 | 357 | 345 | 353 |
| **P1B/C.3** | 345 | 341 | 349 | 349 | 345 | 349 | 353 | 357 | 360 | 360 | 345 | 345 | 349 | 349 | 353 | 357 | 349 | 345 | 353 | 360 |
| **P1B/C.4** | 368 | 357 | 357 | 357 | 357 | 357 | 364 | 364 | 392 | 415 | 353 | 357 | 357 | 349 | 368 | 357 | 353 | 353 | 376 | 411 |
| **P1B/C.5** | 325 | 317 | 317 | 317 | 439 | 364 | 372 | 372 | 376 | 368 | 329 | 325 | 403 | 349 | 403 | 368 | 446 | 411 | 372 | 364 |
| **P1B/C.6** | 431 | 478 | 423 | 431 | 423 | 427 | 423 | 431 | 423 | 431 | 423 | 435 | 423 | 435 | 478 | 435 | 423 | 423 | 431 | 427 |
| **P1B/C.7** | 321 | 341 | 325 | 329 | 317 | 329 | 310 | 317 | 357 | 310 | 317 | 329 | 306 | 329 | 298 | 415 | 403 | 396 | 302 | 298 |
| **P1B/C.8** | 341 | 333 | 337 | 329 | 337 | 337 | 349 | 349 | 360 | 353 | 333 | 298 | 329 | 337 | 345 | 341 | 325 | 333 | 349 | 353 |
| **P1B/C.9** | 368 | 360 | 360 | 360 | 360 | 360 | 349 | 357 | 353 | 364 | 329 | 360 | 368 | 355 | 357 | 353 | 364 | 353 | 349 | 423 |
| **P1B/C.10** | 329 | 345 | 329 | 317 | 329 | 321 | 325 | 310 | 325 | 372 | 324 | 325 | 337 | 321 | 353 | 337 | 321 | 314 | 368 | 392 |
| **P1B/C.11** | 470 | 458 | 407 | 411 | 411 | 403 | 415 | 419 | 415 | 415 | 466 | 474 | 431 | 407 | 423 | 407 | 403 | 411 | 411 | 415 |
| **P1B/C.12** | 364 | 345 | 403 | 400 | 411 | 407 | 407 | 411 | 435 | 427 | 368 | 396 | 415 | 403 | 431 | 415 | 400 | 411 | 415 | 423 |
| **P1B/C.13** | 442 | 442 | 349 | 345 | 349 | 345 | 345 | 353 | 349 | 357 | 345 | 345 | 345 | 345 | 349 | 353 | 345 | 345 | 349 | 357 |
| **P1B/C.14** | 376 | 423 | 407 | 376 | 357 | 392 | 357 | 372 | 360 | 368 | 392 | 407 | 396 | 400 | 349 | 380 | 392 | 360 | 360 | 364 |
| **P1B/C.15** | 392 | 388 | 400 | 388 | 411 | 400 | 427 | 411 | 446 | 435 | 396 | 372 | 415 | 392 | 439 | 392 | 403 | 415 | 411 | 442 |
| **P1B/C.16** | 454 | 458 | 419 | 427 | 400 | 415 | 384 | 400 | 380 | 384 | 423 | 442 | 396 | 411 | 392 | 403 | 415 | 400 | 388 | 376 |
| **P1B/C.17** | 384 | 380 | 364 | 364 | 357 | 357 | 357 | 357 | 349 | 353 | 368 | 360 | 360 | 364 | 360 | 364 | 364 | 357 | 349 | 349 |
| **P1B/C.18** | 470 | 462 | 384 | 450 | 392 | 388 | 392 | 388 | 388 | 392 | 388 | 470 | 392 | 384 | 392 | 392 | 458 | 384 | 388 | 392 |
| **P1B/C.19** | 548 | 544 | 548 | 544 | 548 | 544 | 536 | 528 | 505 | 509 | 548 | 536 | 552 | 544 | 544 | 521 | 548 | 544 | 528 | 513 |
| **P1B/C.20** | 403 | 411 | 439 | 435 | 458 | 458 | 458 | 450 | 458 | 454 | 528 | 544 | 454 | 466 | 466 | 446 | 435 | 446 | 460 | 454 |
| **P1B/C.21** | 388 | 396 | 392 | 400 | 403 | 415 | 423 | 427 | 423 | 435 | 392 | 403 | 407 | 403 | 384 | 423 | 368 | 403 | 419 | 431 |
| **P1B/C.22** | 403 | 415 | 423 | 419 | 423 | 423 | 415 | 419 | 360 | 353 | 435 | 423 | 419 | 427 | 364 | 419 | 423 | 423 | 341 | 349 |
| **P1B/C.23** | 333 | 337 | 360 | 349 | 364 | 345 | 360 | 345 | 349 | 341 | 341 | 321 | 368 | 341 | 364 |  | 349 | 353 | 349 | 345 |
| **P2A** | **Auditory P300 - Latency** | | | | | | | | |  |  |  |  |  |  |  |  |  |  |  |
| **Patient 2A** | **Fp1** | **Fp2** | **F3** | **F4** | **C3** | **C4** | **P3** | **P4** | **O1** | **O2** | **F7** | **F8** | **T3** | **T4** | **T5** | **T6** | **Fz** | **Cz** | **Pz** | **Oz** |
| **P2A.1** | 380 | 435 | 403 | 419 | 415 | 415 | 415 | 419 | 411 | 415 | 400 | 419 | 407 | 419 | 411 | 423 | 419 | 431 | 411 | 423 |
| **P2A.2** | 306 | 310 | 353 | 333 | 357 | 349 | 368 | 368 | 372 | 376 | 341 | 314 | 353 | 341 | 368 | 364 | 341 | 349 | 372 | 376 |
| **P2A.3** | 364 | 360 | 353 | 353 | 349 | 345 | 341 | 337 | 337 | 345 | 353 | 372 | 349 | 345 | 345 | 345 | 349 | 349 | 333 | 333 |
| **P2A.4** | 341 | 341 | 357 | 357 | 345 | 345 | 345 | 325 | 341 | 333 | 360 | 353 | 353 | 329 | 349 | 329 | 353 | 333 | 329 | 341 |
| **P2A.5** | 341 | 360 | 400 | 392 | 392 | 392 | 396 | 380 | 380 | 376 | 411 | 396 | 403 | 388 | 400 | 384 | 392 | 396 | 392 | 376 |
| **P2B/C** | **Auditory P300 - Latency** | | | | | | | | |  |  |  |  |  |  |  |  |  |  |  |
| **Patient 2B/C** | **Fp1** | **Fp2** | **F3** | **F4** | **C3** | **C4** | **P3** | **P4** | **O1** | **O2** | **F7** | **F8** | **T3** | **T4** | **T5** | **T6** | **Fz** | **Cz** | **Pz** | **Oz** |
| **P2B/C.1** | 325 | 357 | 345 | 349 | 357 | 357 | 368 | 372 | 380 | 376 | 341 | 357 | 372 | 368 | 376 | 380 | 345 | 349 | 364 | 376 |
| **P2B/C.2** | 349 | 341 | 357 | 360 | 353 | 357 | 353 | 345 | 345 | 337 | 357 | 353 | 360 | 353 | 364 | 353 | 357 | 353 | 345 | 341 |
| **P2B/C.3** | 376 | 376 | 368 | 368 | 364 | 364 | 321 | 360 | 368 | 368 | 364 | 364 | 364 | 364 | 364 | 360 | 368 | 364 | 364 | 368 |
| **P2B/C.4** | 329 | 317 | 302 | 302 | 310 | 314 | 376 | 321 | 349 | 314 | 302 | 380 | 310 | 306 | 317 | 317 | 302 | 314 | 337 | 341 |
| **P2B/C.5** | 396 | 345 | 337 | 337 | 333 | 329 | 349 | 333 | 384 | 388 | 337 | 333 | 345 | 333 | 349 | 337 | 337 | 329 | 345 | 380 |
| **P2B/C.6** | 341 | 337 | 329 | 325 | 333 | 325 | 337 | 333 | 357 | 384 | 333 | 325 | 341 | 333 | 388 | 333 | 329 | 325 | 337 | 337 |
| **P2B/C.7** | 525 | 552 | 532 | 564 | 540 | 556 | 540 | 540 | 478 | 525 | 525 | 552 | 525 | 548 | 470 | 521 | 567 | 544 | 540 | 513 |
| **P2B/C.8** | 321 | 329 | 341 | 341 | 345 | 341 | 345 | 345 | 353 | 341 | 337 | 337 | 349 | 341 | 349 | 345 | 341 | 341 | 337 | 341 |
| **P2B/C.9** | 357 | 349 | 345 | 345 | 357 | 345 | 360 | 353 | 368 | 364 | 357 | 349 | 376 | 357 | 353 | 357 | 337 | 345 | 357 | 368 |
| **P2B/C.10** | 521 | 513 | 513 | 513 | 517 | 521 | 521 | 525 | 525 | 528 | 509 | 521 | 513 | 536 | 517 | 536 | 513 | 528 | 521 | 525 |
| **P2B/C.11** | 310 | 317 | 325 | 321 | 329 | 321 | 341 | 341 | 345 | 349 | 325 | 329 | 333 | 341 | 345 | 353 | 321 | 325 | 333 | 341 |
| **P2B/C.12** | 493 | 474 | 458 | 462 | 454 | 458 | 349 | 454 | 450 | 446 | 478 | 466 | 470 | 466 | 466 | 462 | 454 | 458 | 458 | 450 |
| **P2B/C.13** | 329 | 337 | 341 | 341 | 345 | 345 | 364 | 345 | 325 | 360 | 329 | 345 | 341 | 345 | 345 | 353 | 337 | 341 | 341 | 317 |
| **P2B/C.14** | 364 | 368 | 380 | 368 | 380 | 380 | 380 | 372 | 376 | 368 | 376 | 364 | 384 | 372 | 384 | 372 | 372 | 380 | 372 | 368 |
| **P2B/C.15** | 337 | 321 | 341 | 333 | 333 | 333 | 388 | 360 | 392 | 388 | 341 | 329 | 396 | 353 | 392 | 364 | 333 | 325 | 345 | 388 |
| **P2B/C.17** | 400 | 403 | 439 | 439 | 501 | 470 | 497 | 482 | 505 | 489 | 396 | 435 | 501 | 474 | 501 | 485 | 446 | 470 | 489 | 501 |
| **P2B/C.18** | 345 | 400 | 376 | 396 | 360 | 396 | 353 | 384 | 470 | 470 | 341 | 360 | 345 | 380 | 337 | 380 | 384 | 368 | 411 | 470 |
| **P2B/C.19** | 341 | 337 | 341 | 337 | 337 | 337 | 349 | 353 | 368 | 357 | 333 | 341 | 341 | 341 | 341 |  | 337 | 345 | 349 | 357 |
| **Control** | **Auditory P300 - Latency** | | | | | | | |  |  |  |  |  |  |  |  |  |  |  |  |
| VIH(-) | **Fp1** | **Fp2** | **F3** | **F4** | **C3** | **C4** | **P3** | **P4** | **O1** | **O2** | **F7** | **F8** | **T3** | **T4** | **T5** | **T6** | **Fz** | **Cz** | **Pz** | **Oz** |
| CNeu.1 | 310 | 306 | 333 | 333 | 337 | 337 | 353 | 364 | 376 | 384 | 310 | 310 | 325 | 290 | 353 | 294 | 333 | 341 | 349 | 357 |
| CNeu.2 | 364 | 353 | 380 | 364 | 360 | 376 | 372 | 345 | 357 | 357 | 388 | 376 | 388 | 384 | 357 | 357 | 357 | 364 | 349 | 348 |
| CNeu.3 | 357 | 384 | 357 | 357 | 345 | 345 | 353 | 392 | 341 | 349 | 400 | 388 | 360 | 364 | 368 | 368 | 349 | 349 | 345 | 355 |
| CNeu.4 | 298 | 306 | 321 | 317 | 341 | 325 | 364 | 337 | 368 | 341 | 286 | 314 | 314 | 321 | 360 | 333 | 314 | 321 | 329 | 338 |
| CNeu.5 | 380 | 380 | 349 | 376 | 317 | 341 | 310 | 325 | 278 | 306 | 333 | 407 | 308 | 411 | 294 | 388 | 337 | 314 | 306 | 315 |
| CNeu.6 | 325 | 329 | 341 | 337 | 341 | 333 | 333 | 325 | 294 | 294 | 345 | 341 | 349 | 337 | 337 | 325 | 333 | 329 | 325 | 328 |
| CNeu.7 | 341 | 337 | 333 | 329 | 329 | 317 | 329 | 317 | 314 | 290 | 337 | 329 | 329 | 321 | 321 | 298 | 321 | 306 | 317 | 320 |
| CNeu.8 | 344 | 342 | 364 | 364 | 364 | 360 | 372 | 364 | 357 | 357 | 396 | 380 | 376 | 376 | 364 | 368 | 357 | 349 | 357 | 357 |
| CNeu.9 | 310 | 321 | 333 | 329 | 325 | 321 | 321 | 317 | 302 | 302 | 349 | 321 | 341 | 317 | 333 | 329 | 321 | 310 | 302 | 310 |
| CNeu.10 | 407 | 407 | 372 | 353 | 333 | 321 | 278 | 290 | 251 | 251 | 376 | 364 | 337 | 317 | 298 | 278 | 360 | 314 | 267 | 375 |
| CNeu.11 | 368 | 360 | 368 | 357 | 357 | 357 | 349 | 353 | 341 | 353 | 376 | 368 | 357 | 360 | 341 | 357 | 353 | 345 | 345 | 345 |
| CNeu.12 | 314 | 317 | 360 | 345 | 333 | 345 | 341 | 345 | 337 | 333 | 314 | 325 | 317 | 329 | 353 | 329 | 341 | 341 | 337 | 329 |
| CNeu.13 | 314 | 314 | 329 | 333 | 329 | 329 | 329 | 329 | 317 | 317 | 329 | 325 | 329 | 321 | 325 | 325 | 337 | 337 | 329 | 306 |
| CNeu.14 | 286 | 247 | 306 | 314 | 294 | 302 | 298 | 294 | 310 | 294 | 298 | 298 | 298 | 302 | 321 | 306 | 317 | 298 | 294 | 310 |
| CNeu.17 | 325 | 321 | 349 | 341 | 357 | 345 | 364 | 349 | 368 | 345 | 317 | 319 | 360 | 329 | 364 | 337 | 345 | 349 | 357 | 360 |
| CNeu.19 | 320 | 357 | 372 | 394 | 376 | 372 | 392 | 376 | 400 | 384 | 360 | 368 | 388 | 392 | 403 | 372 | 372 | 372 | 372 | 368 |
| CNeu.20 | 396 | 364 | 380 | 384 | 376 | 304 | 380 | 376 | 380 | 352 | 380 | 380 | 392 | 396 | 395 | 380 | 376 | 372 | 368 | 364 |
| CNeu.21 | 302 | 294 | 310 | 306 | 325 | 317 | 333 | 325 | 333 | 325 | 298 | 302 | 306 | 317 | 345 | 317 | 314 | 317 | 325 | 329 |
| CNeu.22 | 337 | 325 | 333 | 333 | 337 | 333 | 341 | 337 | 335 | 337 | 343 | 333 | 337 | 342 | 345 | 345 | 337 | 333 | 333 | 337 |
| CNeu.23 | 357 | 353 | 341 | 349 | 367 | 341 | 337 | 341 | 333 | 345 | 314 | 329 | 314 | 304 | 321 | 337 | 337 | 341 | 341 | 341 |
| CNeu.24 | 251 | 263 | 263 | 275 | 271 | 282 | 282 | 282 | 278 | 275 | 237 | 275 | 263 | 275 | 266 | 275 | 275 | 282 | 282 | 278 |
| CNeu.25 | 396 | 372 | 407 | 384 | 407 | 400 | 384 | 380 | 351 | 402 | 400 | 384 | 484 | 384 | 384 | 411 | 443 | 400 | 376 |  |

Mean of latency values at each electrode location (according to the 10/20 International System [40]) are expressed in milliseconds (ms)
