# Supplementary material for: Defects in immune response to Toxoplasma gondii are associated with enhanced HIV-1-related neurocognitive impairment in co-infected patients
Source: PLoS One. 2023 May 24;18(5):e0285976. doi: 10.1371/journal.pone.0285976 (PMC10208516; doi:10.1371/journal.pone.0285976)
Supplement: S5 Table — (DOC) [file pone.0285976.s005.doc]

**S5 Table – Auditory P300 Amplitude**

| **P1A** | **Auditory P300 - Amplitude** | | | | | | | | |  |  |  |  |  |  |  |  |  |  |  |
| --- | --- | --- | --- | --- | --- | --- | --- | --- | --- | --- | --- | --- | --- | --- | --- | --- | --- | --- | --- | --- |
| **Patient 1A** | **Fp1** | **Fp2** | **F3** | **F4** | **C3** | **C4** | **P3** | **P4** | **O1** | **O2** | **F7** | **F8** | **T3** | **T4** | **T5** | **T6** | **Fz** | **Cz** | **Pz** | **Oz** |
| **P1A.1** | 3.9 | 4.8 | 3.4 | 1.7 | 2.2 | 4.0 | 7.4 | 5.5 | 5.3 | 5.0 | 2.5 | 1.2 | 3.4 | 2.9 | 6.5 | 7.1 | 1.3 | 2.8 | 4.6 | 6.0 |
| **P1A.2** | 6.9 | 8.2 | 5.6 | 6.3 | 4.0 | 5.7 | 5.1 | 4.2 | 3.9 | 4.1 | 7.1 | 8.4 | 5.0 | 6.1 | 5.9 | 6.8 | 5.2 | 3.5 | 4.1 | 4.0 |
| **P1A.3** | 13.0 | 7.2 | 8.0 | 5.3 | 10.0 | 8.0 | 13.0 | 11.0 | 9.7 | 10.0 | 11.0 | 4.1 | 9.8 | 4.9 | 12.0 | 8.5 | 7.3 | 9.3 | 12.0 | 11.0 |
| **P1A.4** | 11.0 | 4.2 | 3.0 | 2.1 | 2.3 | 2.6 | 3.2 | 5.7 | 3.2 | 2.6 | 2.9 | 2.4 | 2.5 | 3.6 | 3.2 | 2.8 | 4.9 | 3.2 | 3.1 | 1.5 |
| **P1A.5** | 1.9 | 2.4 | 0.2 | 0.6 | 0.4 | 0.9 | 3.1 | 4.3 | 6.8 | 6.2 | 0.2 | 0.1 | 1.0 | 2.4 | 2.0 | 4.4 | 0.1 | 0.2 | 0.7 | 3.7 |
| **P1A.6** | 6.9 |  | 1.1 | 2.6 | 1.0 | 0.7 | 1.8 | 1.7 | 3.5 | 2.9 |  | 3.4 | 0.9 | 1.2 | 1.0 | 0.2 | 2.5 | 1.9 | 1.6 | 2.9 |
| **P1A.7** | 15.0 | 12.0 | 9.7 | 6.8 | 10.0 | 7.3 | 9.2 | 5.4 | 7.1 | 3.8 | 9.3 | 3.8 | 8.7 | 3.1 | 8.5 | 3.6 | 10.0 | 9.8 | 7.4 | 4.0 |
| **P1A.8** | 3.4 | 1.5 | 2.6 | 1.7 | 3.2 | 2.4 | 4.4 | 2.9 | 4.7 | 3.1 | 3.5 | 1.0 | 3.4 | 2.1 | 3.7 | 2.5 | 2.0 | 2.8 | 4.1 | 4.6 |
| **P1A.9** | 1.5 | 1.3 | 1.8 | 4.0 | 3.6 | 4.1 | 5.8 | 6.5 | 4.2 | 6.3 | 1.9 | 1.9 | 3.1 | 6.1 | 4.6 | 6.7 | 6.3 | 4.5 | 3.6 | 4.5 |
| **P1B/C** | **Auditory P300 - Amplitude** | | | | | | | | |  |  |  |  |  |  |  |  |  |  |  |
| **Patient 1B/C** | **Fp1** | **Fp2** | **F3** | **F4** | **C3** | **C4** | **P3** | **P4** | **O1** | **O2** | **F7** | **F8** | **T3** | **T4** | **T5** | **T6** | **Fz** | **Cz** | **Pz** | **Oz** |
| **P1B/C.1** | 2.3 | 4.9 | 5.4 | 3.2 | 4.6 | 6.8 | 7.0 | 7.2 | 7.4 | 7.7 | 4.2 | 5.4 | 3.8 | 5.4 | 5.7 | 7.2 | 4.7 | 5.5 | 5.9 | 8.7 |
| **P1B/C.2** | 1.0 | 0.2 | 0.3 | 0.6 | 0.2 | 0.6 | 2.4 | 1.1 | 0.7 | 1.4 | 1.0 | 0.9 | 0.8 | 0.1 | 0.9 | 1.6 | 0.1 | 0.9 | 0.9 | 0.7 |
| **P1B/C.3** | 1.2 | 0.2 | 5.8 | 2.7 | 9.0 | 7.0 | 9.8 | 7.9 | 9.8 | 7.0 | 4.4 | 0.5 | 8.5 | 4.7 | 9.9 | 5.3 | 3.3 | 13.0 | 10.0 | 6.6 |
| **P1B/C.4** | 3.3 | 5.4 | 3.6 | 3.6 | 3.7 | 3.0 | 3.1 | 2.9 | 2.8 | 1.8 | 2.5 | 3.1 | 2.9 | 4.0 | 4.4 | 3.5 | 3.5 | 3.0 | 2.9 | 2.0 |
| **P1B/C.5** | 0.4 | 2.3 | 0.5 | 0.5 | 0.2 | 0.5 | 5.6 | 5.3 | 4.9 | 1.4 | 1.4 | 3.7 | 4.3 | 2.3 | 5.4 | 4.1 | 0.3 | 0.8 | 5.8 | 6.7 |
| **P1B/C.6** | 2.1 | 0.3 | 0.0 | 2.4 | 5.4 | 3.6 | 7.0 | 5.2 | 5.6 | 1.0 | 1.0 | 5.7 | 5.2 | 4.3 | 1.2 | 4.9 | 3.9 | 3.7 | 5.6 | 7.1 |
| **P1B/C.7** | 3.1 | 1.7 | 4.2 | 2.6 | 5.1 | 3.5 | 5.2 | 4.6 | 5.1 | 3.9 | 3.9 | 0.9 | 6.6 | 3.0 | 6.5 | 0.3 | 1.7 | 5.2 | 4.4 | 5.7 |
| **P1B/C.8** | 1.1 | 4.5 | 7.0 | 9.6 | 8.1 | 8.5 | 5.5 | 7.8 | 8.1 | 7.6 | 4.6 | 7.6 | 5.8 | 7.8 | 9.6 | 9.1 | 7.1 | 8.5 | 7.5 | 7.0 |
| **P1B/C.9** | 2.7 | 2.8 | 5.1 | 1.0 | 3.6 | 2.3 | 2.9 | 2.6 | 3.5 | 2.1 | 1.7 | 0.1 | 5.1 | 3.3 | 1.8 | 3.3 | 3.4 | 3.2 | 3.9 | 3.3 |
| **P1B/C.10** | 7.3 | 1.5 | 0.0 | 3.0 | 1.0 | 3.8 | 2.2 | 1.5 | 1.7 | 2.4 | 1.4 | 2.7 | 2.2 | 3.7 | 3.5 | 3.9 | 1.3 | 0.1 | 1.6 | 1.2 |
| **P1B/C.11** | 6.7 | 5.9 | 4.3 | 4.7 | 5.5 | 5.6 | 5.7 | 1.0 | 4.1 | 4.1 | 4.3 | 3.4 | 4.3 | 4.8 | 5.3 | 5.3 | 4.3 | 5.4 | 5.1 | 4.3 |
| **P1B/C.12** | 8.6 | 0.9 | 3.2 | 0.6 | 1.5 | 3.3 | 4.1 | 2.6 | 0.9 | 1.9 | 3.9 | 0.3 | 2.1 | 0.1 | 1.8 | 0.3 | 3.6 | 2.7 | 4.2 | 2.3 |
| **P1B/C.13** | 9.3 | 8.0 | 7.8 | 11.0 | 12.0 | 9.9 | 12.0 | 11.0 | 7.5 | 10.0 | 3.9 | 6.3 | 9.1 | 8.9 | 8.6 | 11.0 | 9.0 | 12.0 | 12.0 | 9.2 |
| **P1B/C.14** | 4.3 | 2.3 | 1.2 | 0.7 | 1.9 | 1.8 | 0.0 | 0.2 | 2.4 | 1.3 | 3.2 | 0.4 | 0.1 | 1.3 | 1.4 | 2.3 | 1.4 | 1.9 | 1.0 | 2.3 |
| **P1B/C.15** | 0.4 | 0.2 | 1.8 | 1.7 | 2.9 | 4.3 | 3.6 | 4.4 | 4.1 | 2.4 | 1.8 | 1.8 | 1.7 | 2.3 | 5.3 | 3.1 | 2.7 | 2.2 | 5.5 | 3.5 |
| **P1B/C.16** | 17.0 | 23.0 | 11.0 | 11.0 | 7.8 | 10.0 | 8.5 | 8.1 | 5.2 | 8.8 | 10.0 | 11.0 | 10.0 | 8.5 | 6.5 | 8.6 | 8.6 | 7.8 | 10.0 | 6.3 |
| **P1B/C.17** | 2.6 | 3.2 | 3.0 | 4.5 | 4.8 | 5.1 | 3.8 | 3.9 | 3.6 | 2.1 | 0.5 | 2.1 | 2.9 | 2.5 | 3.9 | 3.6 | 4.3 | 5.7 | 4.7 | 3.1 |
| **P1B/C.18** | 1.8 | 5.7 | 0.2 | 4.7 | 2.4 | 0.8 | 5.5 | 9.1 | 3.3 | 5.0 | 0.1 | 1.1 | 3.9 | 2.1 | 3.2 | 5.5 | 0.6 | 1.8 | 4.6 | 5.9 |
| **P1B/C.19** | 7.1 | 8.2 | 12.0 | 12.0 | 11.0 | 11.0 | 7.0 | 8.8 | 6.1 | 5.3 | 6.5 | 11.0 | 6.1 | 8.1 | 5.3 | 6.3 | 11.0 | 10.0 | 6.5 | 6.7 |
| **P1B/C.20** | 2.9 | 1.3 | 0.2 | 0.8 | 3.9 | 4.6 | 6.6 | 5.7 | 5.6 | 4.6 | 1.6 | 0.7 | 2.3 | 2.7 | 4.5 | 4.1 | 0.1 | 2.9 | 4.7 | 4.5 |
| **P1B/C.21** | 2.8 | 1.0 | 2.0 | 1.4 | 0.5 | 0.3 | 2.5 | 1.9 | 2.9 | 0.8 | 0.8 | 1.4 | 2.4 | 1.3 | 3.0 | 1.8 | 0.6 | 1.4 | 1.9 | 2.7 |
| **P1B/C.22** | 4.5 | 4.9 | 4.4 | 5.0 | 4.9 | 5.4 | 5.4 | 5.4 | 2.4 | 5.0 | 2.5 | 4.9 | 3.8 | 5.8 | 2.8 | 4.6 | 4.8 | 4.5 | 5.2 | 3.8 |
| **P1B/C.23** | 0.7 | 6.6 | 3.5 | 4.7 | 4.7 | 5.6 | 6.7 | 7.4 | 2.7 | 0.5 | 1.2 | 2.2 | 4.4 | 4.9 | 5.2 | 6.4 | 4.1 | 5.0 | 2.7 | 5.1 |
| **P2A** | **Auditory P300 - Amplitude** | | | | | | | | |  |  |  |  |  |  |  |  |  |  |  |
| **Patient 2A** | **Fp1** | **Fp2** | **F3** | **F4** | **C3** | **C4** | **P3** | **P4** | **O1** | **O2** | **F7** | **F8** | **T3** | **T4** | **T5** | **T6** | **Fz** | **Cz** | **Pz** | **Oz** |
| **P2A.1** | 0.6 | 0.7 | 3.8 | 4.2 | 4.7 | 7.4 | 5.4 | 5.9 | 4.3 | 2.6 | 4.6 | 2.4 | 5.0 | 4.1 | 4.9 | 4.0 | 2.6 | 3.0 | 3.4 | 5.9 |
| **P2A.2** | 3.6 | 5.1 | 1.5 | 1.4 | 3.8 | 3.9 | 7.1 | 5.0 | 8.5 | 5.8 | 4.4 | 5.2 | 3.4 | 2.7 | 7.7 | 4.8 | 1.8 | 3.1 | 6.1 | 6.7 |
| **P2A.3** | 2.9 | 4.3 | 0.5 | 1.7 | 1.7 | 0.2 | 4.6 | 3.6 | 7.1 | 4.5 | 4.0 | 0.9 | 2.2 | 0.3 | 5.3 | 2.9 | 1.1 | 2.0 | 3.6 | 3.4 |
| **P2A.4** | 0.5 | 3.0 | 5.2 | 3.8 | 6.2 | 6.8 | 8.4 | 9.4 | 9.9 | 2.6 | 3.8 | 4.0 | 6.7 | 5.0 | 8.4 | 7.1 | 5.3 | 6.1 | 9.4 | 11.0 |
| **P2A.5** | 3.1 | 1.0 | 1.4 | 2.2 | 0.3 | 0.4 | 2.9 | 2.6 | 4.3 | 1.7 | 0.6 | 1.9 | 2.2 | 1.7 | 0.7 | 1.7 | 2.0 | 1.5 | 3.3 | 3.6 |
| **P2B/C** | **Auditory P300 - Amplitude** | | | | | | | | |  |  |  |  |  |  |  |  |  |  |  |
| **Patient 2B/C** | **Fp1** | **Fp2** | **F3** | **F4** | **C3** | **C4** | **P3** | **P4** | **O1** | **O2** | **F7** | **F8** | **T3** | **T4** | **T5** | **T6** | **Fz** | **Cz** | **Pz** | **Oz** |
| **P2B/C.1** | 2.6 | 2.8 | 7.6 | 5.9 | 4.2 | 4.0 | 4.2 | 5.0 | 4.9 | 5.0 | 3.8 | 3.5 | 3.0 | 4.3 | 4.5 | 4.8 | 6.1 | 4.9 | 4.9 | 4.2 |
| **P2B/C.2** | 2.4 | 5.3 | 1.1 | 3.0 | 0.6 | 2.8 | 2.5 | 2.8 | 2.4 | 3.4 | 1.1 | 4.0 | 2.2 | 2.9 | 2.9 | 3.3 | 2.5 | 1.4 | 2.5 | 2.4 |
| **P2B/C.3** | 1.2 | 1.5 | 2.4 | 3.8 | 4.9 | 4.8 | 6.4 | 5.4 | 6.8 | 5.4 | 1.0 | 2.7 | 2.4 | 3.3 | 5.8 | 4.6 | 3.8 | 6.6 | 6.7 | 6.2 |
| **P2B/C.4** | 3.5 | 3.2 | 1.2 | 4.3 | 2.2 | 3.9 | 3.2 | 2.7 | 4.5 | 1.3 | 0.9 | 1.8 | 0.9 | 2.9 | 2.0 | 2.2 | 3.6 | 3.4 | 3.2 | 3.2 |
| **P2B/C.5** | 0.7 | 4.2 | 6.8 | 13.0 | 7.8 | 8.8 | 10.0 | 8.9 | 8.0 | 6.4 | 15.0 | 10.0 | 5.3 | 8.0 | 11.0 | 6.4 | 13.0 | 7.8 | 11.0 | 11.0 |
| **P2B/C.6** | 6.4 | 6.5 | 5.5 | 5.4 | 4.2 | 3.8 | 4.3 | 4.1 | 3.3 | 2.4 | 4.2 | 3.3 | 5.2 | 4.5 | 4.7 | 3.8 | 5.4 | 4.3 | 4.1 | 2.3 |
| **P2B/C.7** | 2.4 | 2.8 | 2.3 | 1.3 | 1.6 | 2.5 | 1.7 | 2.1 | 1.6 | 5.2 | 2.6 | 3.0 | 1.3 | 1.6 | 0.7 | 1.6 | 0.9 | 2.1 | 2.6 | 1.2 |
| **P2B/C.8** | 2.4 | 3.8 | 5.8 | 5.4 | 4.5 | 5.5 | 5.5 | 5.6 | 5.3 | 5.7 | 4.1 | 5.5 | 3.6 |  | 4.5 | 5.5 | 5.8 | 5.3 | 6.2 | 5.2 |
| **P2B/C.9** | 10.0 | 6.0 | 2.5 | 4.2 | 2.8 | 3.9 | 3.6 | 4.3 | 6.9 | 2.4 | 2.4 | 2.4 | 2.1 | 3.3 | 4.2 | 5.5 | 3.3 | 2.3 | 3.9 | 5.9 |
| **P2B/C.10** | 2.7 | 5.6 | 3.3 | 2.8 | 4.3 | 4.1 | 5.4 | 4.4 | 2.9 | 3.4 | 2.1 | 2.7 | 5.5 | 2.9 | 4.1 | 1.9 | 2.4 | 0.9 | 4.6 | 3.0 |
| **P2B/C.11** | 0.1 | 0.0 | 3.1 | 4.8 | 6.4 | 4.9 | 7.7 | 7.4 | 10.0 | 8.0 | 3.1 | 3.2 | 7.4 | 4.4 | 7.3 | 8.7 | 4.5 | 5.5 | 7.4 | 8.4 |
| **P2B/C.12** | 0.7 | 1.7 | 3.6 | 3.7 | 3.8 | 4.1 | 4.7 | 5.3 | 5.9 | 5.6 | 0.3 | 3.2 | 2.4 | 3.5 | 4.3 | 4.4 | 5.0 | 4.2 | 6.1 | 6.1 |
| **P2B/C.13** | 12.0 | 16.0 | 9.7 | 8.2 | 5.6 | 9.0 | 9.5 | 9.5 | 8.0 | 6.4 | 12.0 | 11.0 | 7.4 | 6.6 | 8.0 | 7.8 | 9.4 | 8.8 | 9.1 | 7.5 |
| **P2B/C.14** | 2.8 | 5.1 | 4.0 | 3.5 | 5.8 | 5.1 | 6.6 | 5.0 | 4.3 | 5.3 | 3.1 | 4.1 | 4.3 | 3.5 | 5.5 | 4.3 | 3.5 | 4.3 | 6.1 | 5.2 |
| **P2B/C.15** | 2.8 | 6.0 | 1.8 | 1.8 | 1.0 | 2.3 | 2.1 | 3.7 | 4.4 | 4.4 | 2.1 | 1.4 | 1.4 | 3.9 | 2.9 | 3.9 | 1.4 | 2.0 | 3.6 | 4.7 |
| **P2B/C.17** | 3.7 | 0.8 | 0.2 | 1.6 | 1.4 | 0.4 | 1.9 | 0.9 | 2.3 | 1.5 | 1.1 | 0.2 | 1.8 | 0.1 | 2.0 | 0.5 | 0.2 | 1.2 | 1.7 | 1.5 |
| **P2B/C.18** | 3.4 | 3.7 | 3.6 | 4.7 | 1.2 | 6.0 | 0.2 | 2.1 | 1.9 | 2.2 | 1.5 | 3.0 | 1.1 | 3.0 | 0.1 | 2.7 | 3.7 | 0.9 | 1.0 | 1.4 |
| **P2B/C.19** | 1.2 | 1.0 | 3.6 | 4.3 | 5.1 | 4.5 | 5.8 | 4.6 | 4.3 | 4.5 | 3.2 | 4.2 | 1.6 | 2.0 | 4.0 | 2.6 | 2.5 | 5.2 | 6.4 | 3.4 |
| **Control** | **Auditory P300 - Amplitude** | | | | | | | |  |  |  |  |  |  |  |  |  |  |  |  |
| **VIH(-)** | **Fp1** | **Fp2** | **F3** | **F4** | **C3** | **C4** | **P3** | **P4** | **O1** | **O2** | **F7** | **F8** | **T3** | **T4** | **T5** | **T6** | **Fz** | **Cz** | **Pz** | **Oz** |
| CNeu.1 | 4.9 | 3.9 | 5.0 | 5.2 | 1.6 | 3.0 | 1.1 | 0.9 | 0.3 | 0.3 | 3.4 | 3.0 | 2.3 | 2.1 | 0.6 | 3.3 | 5.0 | 4.8 | 1.2 | 3.8 |
| CNeu.2 | 4.1 | 5.0 | 6.5 | 7.1 | 5.5 | 4.5 | 5.1 | 3.6 | 3.5 | 1.5 | 3.7 | 4.7 | 4.7 | 3.9 | 9.4 | 4.3 | 6.5 | 6.4 | 4.7 | 5.1 |
| CNeu.3 | 7.4 | 7.7 | 6.8 | 8.5 | 10.0 | 10.0 | 7.7 | 10.0 | 1.6 | 3.8 | 0.9 | 3.6 | 5.7 | 7.4 | 1.9 | 1.9 | 10.0 | 13.0 | 8.4 | 8.1 |
| CNeu.4 | 4.1 | 5.5 | 3.1 | 6.6 | 3.8 | 7.7 | 8.7 | 7.9 | 6.1 | 5.9 | 5.2 | 5.0 | 3.0 | 5.7 | 3.6 | 6.0 | 5.6 | 6.2 | 6.5 | 6.5 |
| CNeu.5 | 9.4 | 15.0 | 12.0 | 12.0 | 13.0 | 13.0 | 13.0 | 13.0 | 8.3 | 8.1 | 6.0 | 8.1 | 8.8 | 9.6 | 9.3 | 8.7 | 11.0 | 13.0 | 13.0 | 14.0 |
| CNeu.6 | 15.0 | 17.0 | 13.0 | 15.0 | 11.0 | 14.0 | 9.6 | 12.0 | 6.0 | 6.0 | 7.5 | 9.5 | 6.8 | 9.7 | 4.1 | 8.1 | 5.8 | 14.0 | 13.0 | 11.0 |
| CNeu.7 | 2.7 | 2.3 | 4.2 | 6.1 | 6.4 | 8.4 | 9.5 | 9.5 | 5.4 | 5.4 | 3.5 | 4.3 | 6.8 | 6.6 | 5.3 | 4.8 | 6.3 | 7.5 | 8.5 | 7.6 |
| CNeu.8 | 2.4 | 3.2 | 0.9 | 0.9 | 2.3 | 1.9 | 5.0 | 2.9 | 4.4 | 4.1 | 5.2 | 2.0 | 2.4 | 3.1 | 3.5 | 3.2 | 2.3 | 2.8 | 3.1 | 4.2 |
| CNeu.9 | 1.9 | 1.8 | 6.3 | 6.3 | 10.0 | 12.0 | 11.0 | 9.5 | 4.5 | 5.6 | 2.5 | 3.5 | 5.8 | 6.7 | 8.0 | 4.3 | 5.4 | 10.0 | 9.8 | 8.6 |
| CNeu.10 | 5.8 | 6.2 | 5.4 | 5.5 | 4.4 | 4.8 | 4.2 | 4.4 | 4.7 | 4.7 | 4.9 | 3.1 | 2.6 | 2.2 | 1.1 | 4.0 | 6.6 | 5.5 | 4.4 | 5.6 |
| CNeu.11 | 3.4 | 4.4 | 7.3 | 9.1 | 8.9 | 11.0 | 8.6 | 11.0 | 7.3 | 8.5 | 5.1 | 6.2 | 7.8 | 7.2 | 3.7 | 8.1 | 9.6 | 11.0 | 8.5 | 9.5 |
| CNeu.12 | 12.0 | 8.6 | 3.4 | 4.9 | 3.4 | 3.0 | 3.3 | 1.6 | 2.7 | 2.7 | 7.1 | 5.2 | 4.6 | 2.8 | 5.4 | 2.5 | 5.6 | 3.8 | 2.6 | 3.1 |
| CNeu.13 | 3.8 | 3.8 | 4.6 | 9.2 | 7.5 | 7.7 | 8.6 | 7.2 | 5.6 | 5.9 | 5.1 | 4.0 | 5.6 | 5.8 | 7.8 | 5.3 | 9.6 | 7.3 | 8.0 | 6.3 |
| CNeu.14 | 4.3 | 4.3 | 4.1 | 5.7 | 1.8 | 3.8 | 1.8 | 3.9 | 2.2 | 3.0 | 1.8 | 1.8 | 2.0 | 3.9 | 2.4 | 3.8 | 4.9 | 3.7 | 3.7 | 3.1 |
| CNeu.17 | 1.8 | 3.1 | 6.2 | 7.5 | 7.7 | 11.0 | 8.1 | 12.0 | 7.1 | 7.8 | 2.5 | 2.6 | 3.8 | 8.1 | 5.3 | 7.8 | 7.2 | 10.0 | 11.0 | 9.4 |
| CNeu.19 | 7.5 | 2.0 | 0.7 | 2.7 | 0.6 | 1.1 | 0.3 | 0.5 | 0.1 | 2.6 | 0.7 | 1.2 | 3.2 | 3.3 | 3.7 | 1.8 | 1.1 | 0.1 | 0.1 | 0.4 |
| CNeu.20 | 2.6 | 3.7 | 2.1 | 3.8 | 9.7 | 9.7 | 3.0 | 0.6 | 2.8 | 2.5 | 3.0 | 4.4 | 3.2 | 2.6 | 0.1 | 2.0 | 0.9 | 0.1 | 0.1 | 0.6 |
| CNeu.21 | 2.9 | 0.2 | 2.1 | 3.4 | 1.1 | 2.6 | 2.4 | 2.2 | 3.7 | 2.6 | 0.7 | 0.1 | 0.2 | 1.8 | 1.9 | 1.7 | 2.5 | 2.2 | 1.9 | 2.7 |
| CNeu.22 | 1.8 | 0.9 | 4.4 | 2.3 | 2.5 | 2.3 | 2.8 | 0.8 | 0.7 | 0.3 | 0.4 | 1.9 | 0.5 | 1.4 | 3.4 | 0.9 | 3.9 | 2.1 | 1.1 | 0.4 |
| CNeu.23 | 4.1 | 3.1 | 6.3 | 2.6 | 2.6 | 2.0 | 2.5 | 2.1 | 7.8 | 7.0 | 3.0 | 7.5 | 5.5 | 3.6 | 7.4 | 9.1 | 8.5 | 9.1 | 9.8 | 7.6 |
| CNeu.24 | 1.3 | 3.4 | 4.2 | 6.3 | 4.2 | 6.1 | 5.0 | 8.3 | 8.2 | 10.0 | 2.0 | 4.1 | 3.5 | 5.7 | 3.8 | 8.3 | 4.4 | 5.3 | 7.8 | 9.5 |
| CNeu.25 | 2.7 | 2.1 | 0.4 | 1.3 | 2.6 | 3.1 | 3.8 | 3.8 | 2.5 | 4.2 | 0.7 | 1.2 | 0.3 | 1.4 | 2.2 | 2.7 | 0.4 | 2.2 | 2.4 | 4.5 |

Mean of amplitude values at each electrode location (according to the 10/20 International System [40]) are expressed in microVolts (μV)
