# Supplementary material for: Defects in immune response to Toxoplasma gondii are associated with enhanced HIV-1-related neurocognitive impairment in co-infected patients
Source: PLoS One. 2023 May 24;18(5):e0285976. doi: 10.1371/journal.pone.0285976 (PMC10208516; doi:10.1371/journal.pone.0285976)
Supplement: S6 Table — (DOC) [file pone.0285976.s006.doc]

**S6 Table – Exploration of Memory - Sternberg**

| **P1A** | **Exploration of Memory - Sternberg** | | | | | | | | | | | | | | | | | | | | | | | | | | | | | | | | |  | | |  | | |  | | |  | | |  | | |  | | |  | | |  | | | |  | | | |  | | | |  | | | |  | | | |  | | | |  | |
| --- | --- | --- | --- | --- | --- | --- | --- | --- | --- | --- | --- | --- | --- | --- | --- | --- | --- | --- | --- | --- | --- | --- | --- | --- | --- | --- | --- | --- | --- | --- | --- | --- | --- | --- | --- | --- | --- | --- | --- | --- | --- | --- | --- | --- | --- | --- | --- | --- | --- | --- | --- | --- | --- | --- | --- | --- | --- | --- | --- | --- | --- | --- | --- | --- | --- | --- | --- | --- | --- | --- | --- | --- | --- | --- | --- | --- | --- | --- | --- | --- |
| Patient 1A | TRA | | | TWA | | | TAA | | | RA2 | | | WA2 | | | AA2 | | | RA3 | | | WA3 | | | AA3 | | | RA4 | | | WA4 | | | AA4 | | | RA5 | | | WA5 | | | AA5 | | | RA6 | | | WA6 | | | AA6 | | | TTA | | | | TA2 | | | | TA3 | | | | TA4 | | | | TA5 | | | | TA6 | | | |  | |
| **P1A.1** | 8 | | | 1 | | | 6 | | | 2 | | | 0 | | | 1 | | | 1 | | | 1 | | | 1 | | | 1 | | | 0 | | | 2 | | | 2 | | | 0 | | | 1 | | | 2 | | | 0 | | | 1 | | | 729 | | | | 728 | | | | 593 | | | | 701 | | | | 706 | | | | 834 | | | |  | |
| **P1A.2** | 7 | | | 1 | | | 7 | | | 2 | | | 0 | | | 1 | | | 2 | | | 0 | | | 1 | | | 2 | | | 0 | | | 1 | | | 0 | | | 1 | | | 2 | | | 1 | | | 0 | | | 2 | | | 611 | | | | 564 | | | | 593 | | | | 610 | | | | 2000 | | | | 741 | | | |  | |
| **P1A.3** | 15 | | | 0 | | | 0 | | | 3 | | | 0 | | | 0 | | | 3 | | | 0 | | | 0 | | | 3 | | | 0 | | | 0 | | | 3 | | | 0 | | | 0 | | | 3 | | | 0 | | | 0 | | | 778 | | | | 577 | | | | 796 | | | | 810 | | | | 795 | | | | 914 | | | |  | |
| **P1A.4** | 7 | | | 2 | | | 6 | | | 2 | | | 1 | | | 0 | | | 1 | | | 0 | | | 2 | | | 1 | | | 1 | | | 1 | | | 1 | | | 0 | | | 2 | | | 2 | | | 0 | | | 1 | | | 496 | | | | 437 | | | | 426 | | | | 603 | | | | 578 | | | | 482 | | | |  | |
| **P1A.5** | 11 | | | 2 | | | 2 | | | 2 | | | 0 | | | 1 | | | 3 | | | 0 | | | 0 | | | 3 | | | 0 | | | 0 | | | 2 | | | 1 | | | 0 | | | 1 | | | 1 | | | 1 | | | 1068 | | | | 873 | | | | 1023 | | | | 1056 | | | | 1369 | | | | 1027 | | | |  | |
| **P1A.6** | 3 | | | 0 | | | 12 | | | 0 | | | 0 | | | 3 | | | 0 | | | 0 | | | 3 | | | 2 | | | 0 | | | 1 | | | 0 | | | 0 | | | 3 | | | 1 | | | 0 | | | 2 | | | 866 | | | | 2000 | | | | 2000 | | | | 923 | | | | 2000 | | | | 751 | | | |  | |
| **P1A.7** | 14 | | | 1 | | | 0 | | | 2 | | | 1 | | | 0 | | | 3 | | | 0 | | | 0 | | | 3 | | | 0 | | | 0 | | | 3 | | | 0 | | | 0 | | | 3 | | | 0 | | | 0 | | | 755 | | | | 642 | | | | 781 | | | | 776 | | | | 763 | | | | 779 | | | |  | |
| **P1A.8** | 12 | | | 1 | | | 2 | | | 3 | | | 0 | | | 0 | | | 3 | | | 0 | | | 0 | | | 1 | | | 1 | | | 1 | | | 3 | | | 0 | | | 0 | | | 2 | | | 0 | | | 1 | | | 865 | | | | 583 | | | | 1004 | | | | 934 | | | | 905 | | | | 986 | | | |  | |
| **P1A.9** | 8 | | | 0 | | | 7 | | | 2 | | | 0 | | | 1 | | | 2 | | | 0 | | | 1 | | | 2 | | | 0 | | | 1 | | | 0 | | | 0 | | | 3 | | | 2 | | | 0 | | | 1 | | | 1145 | | | | 1015 | | | | 1293 | | | | 1162 | | | | 2000 | | | | 1109 | | | |  | |
| **P1B/C** | | **Exploration of Memory - Sternberg** | | | | | | | | | | | | | | | | | | | | | | | | | | | | | | | | |  | | |  | | |  | | |  | | |  | | |  | | |  | | |  | | | |  | | | |  | | | |  | | | |  | | | |  | | | |  |
| Patient 1B/C | | TRA | | | TWA | | | TAA | | | RA2 | | | WA2 | | | AA2 | | | RA3 | | | WA3 | | | AA3 | | | RA4 | | | WA4 | | | AA4 | | | RA5 | | | WA5 | | | AA5 | | | RA6 | | | WA6 | | | AA6 | | | TTA | | | | TA2 | | | | TA3 | | | | TA4 | | | | TA5 | | | | TA6 | | | |  |
| **P1B/C.1** | | 9 | | | 1 | | | 5 | | | 2 | | | 0 | | | 1 | | | 3 | | | 0 | | | 0 | | | 1 | | | 0 | | | 2 | | | 2 | | | 0 | | | 1 | | | 1 | | | 1 | | | 1 | | | 902 | | | | 621 | | | | 1084 | | | | 983 | | | | 1004 | | | | 635 | | | |  |
| **P1B/C.2** | | 9 | | | 4 | | | 2 | | | 3 | | | 0 | | | 0 | | | 2 | | | 0 | | | 1 | | | 2 | | | 1 | | | 0 | | | 1 | | | 2 | | | 0 | | | 1 | | | 1 | | | 1 | | | 750 | | | | 741 | | | | 788 | | | | 663 | | | | 919 | | | | 705 | | | |  |
| **P1B/C.3** | | 11 | | | 3 | | | 1 | | | 2 | | | 1 | | | 0 | | | 2 | | | 1 | | | 0 | | | 3 | | | 0 | | | 0 | | | 2 | | | 1 | | | 0 | | | 2 | | | 0 | | | 1 | | | 1097 | | | | 872 | | | | 1349 | | | | 974 | | | | 869 | | | | 1485 | | | |  |
| **P1B/C.4** | | 10 | | | 5 | | | 0 | | | 2 | | | 1 | | | 0 | | | 2 | | | 1 | | | 0 | | | 2 | | | 1 | | | 0 | | | 2 | | | 1 | | | 0 | | | 2 | | | 1 | | | 0 | | | 478 | | | | 550 | | | | 404 | | | | 377 | | | | 487 | | | | 575 | | | |  |
| **P1B/C.5** | | 6 | | | 0 | | | 9 | | | 1 | | | 0 | | | 2 | | | 1 | | | 0 | | | 2 | | | 1 | | | 0 | | | 2 | | | 1 | | | 0 | | | 2 | | | 2 | | | 0 | | | 1 | | | 784 | | | | 1022 | | | | 987 | | | | 553 | | | | 431 | | | | 855 | | | |  |
| **P1B/C.6** | | 2 | | | 0 | | | 13 | | | 0 | | | 0 | | | 3 | | | 1 | | | 0 | | | 2 | | | 0 | | | 0 | | | 3 | | | 1 | | | 0 | | | 2 | | | 0 | | | 0 | | | 3 | | | 1280 | | | | 2000 | | | | 1583 | | | | 2000 | | | | 977 | | | | 2000 | | | |  |
| **P1B/C.7** | | 8 | | | 2 | | | 5 | | | 1 | | | 1 | | | 1 | | | 2 | | | 0 | | | 1 | | | 2 | | | 0 | | | 1 | | | 2 | | | 0 | | | 1 | | | 1 | | | 1 | | | 1 | | | 675 | | | | 612 | | | | 592 | | | | 624 | | | | 739 | | | | 876 | | | |  |
| **P1B/C.8** | | 14 | | | 0 | | | 1 | | | 3 | | | 0 | | | 0 | | | 3 | | | 0 | | | 0 | | | 3 | | | 0 | | | 0 | | | 3 | | | 0 | | | 0 | | | 2 | | | 0 | | | 1 | | | 902 | | | | 798 | | | | 894 | | | | 897 | | | | 869 | | | | 1130 | | | |  |
| **P1B/C.9** | | 11 | | | 4 | | | 0 | | | 2 | | | 1 | | | 0 | | | 3 | | | 0 | | | 0 | | | 3 | | | 0 | | | 0 | | | 1 | | | 2 | | | 0 | | | 2 | | | 1 | | | 0 | | | 803 | | | | 674 | | | | 748 | | | | 740 | | | | 1049 | | | | 987 | | | |  |
| **P1B/C.10** | | 6 | | | 4 | | | 5 | | | 1 | | | 0 | | | 2 | | | 2 | | | 0 | | | 1 | | | 1 | | | 1 | | | 1 | | | 2 | | | 1 | | | 0 | | | 0 | | | 2 | | | 1 | | | 570 | | | | 544 | | | | 487 | | | | 563 | | | | 671 | | | | 2000 | | | |  |
| **P1B/C.11** | | 1 | | | 0 | | | 14 | | | 0 | | | 0 | | | 3 | | | 1 | | | 0 | | | 2 | | | 0 | | | 0 | | | 3 | | | 0 | | | 0 | | | 3 | | | 0 | | | 0 | | | 3 | | | 456 | | | | 2000 | | | | 457 | | | | 2000 | | | | 2000 | | | | 2000 | | | |  |
| **P1B/C.12** | | 11 | | | 4 | | | 0 | | | 2 | | | 1 | | | 0 | | | 2 | | | 1 | | | 0 | | | 3 | | | 0 | | | 0 | | | 2 | | | 1 | | | 0 | | | 2 | | | 1 | | | 0 | | | 530 | | | | 566 | | | | 490 | | | | 588 | | | | 487 | | | | 491 | | | |  |
| **P1B/C.13** | | 7 | | | 2 | | | 6 | | | 2 | | | 0 | | | 1 | | | 1 | | | 0 | | | 2 | | | 2 | | | 1 | | | 0 | | | 1 | | | 1 | | | 1 | | | 1 | | | 0 | | | 2 | | | 550 | | | | 487 | | | | 525 | | | | 595 | | | | 677 | | | | 484 | | | |  |
| **P1B/C.15** | | 8 | | | 7 | | | 0 | | | 2 | | | 1 | | | 0 | | | 1 | | | 2 | | | 0 | | | 1 | | | 2 | | | 0 | | | 2 | | | 1 | | | 0 | | | 2 | | | 1 | | | 0 | | | 872 | | | | 782 | | | | 835 | | | | 538 | | | | 1289 | | | | 730 | | | |  |
| **P1B/C.16** | | 5 | | | 1 | | | 9 | | | 3 | | | 0 | | | 0 | | | 1 | | | 0 | | | 2 | | | 0 | | | 0 | | | 3 | | | 0 | | | 0 | | | 3 | | | 1 | | | 1 | | | 1 | | | 905 | | | | 903 | | | |  | | | | 2000 | | | | 2000 | | | | 709 | | | |  |
| **P1B/C.17** | | 11 | | | 0 | | | 4 | | | 3 | | | 0 | | | 0 | | | 2 | | | 0 | | | 1 | | | 2 | | | 0 | | | 1 | | | 2 | | | 0 | | | 1 | | | 2 | | | 0 | | | 1 | | | 878 | | | | 915 | | | | 958 | | | | 722 | | | | 1047 | | | | 730 | | | |  |
| **P1B/C.18** | | 4 | | | 3 | | | 8 | | | 1 | | | 1 | | | 1 | | | 1 | | | 0 | | | 2 | | | 1 | | | 0 | | | 2 | | | 1 | | | 1 | | | 1 | | | 0 | | | 1 | | | 2 | | | 1277 | | | | 1033 | | | | 1013 | | | | 1170 | | | | 1893 | | | | 2000 | | | |  |
| **P1B/C.19** | | 14 | | | 1 | | | 0 | | | 3 | | | 0 | | | 0 | | | 3 | | | 0 | | | 0 | | | 3 | | | 0 | | | 0 | | | 3 | | | 0 | | | 0 | | | 2 | | | 1 | | | 0 | | | 755 | | | | 678 | | | | 596 | | | | 805 | | | | 874 | | | | 855 | | | |  |
| **P1B/C.20** | | 3 | | | 1 | | | 11 | | | 0 | | | 1 | | | 2 | | | 0 | | | 0 | | | 3 | | | 0 | | | 0 | | | 3 | | | 1 | | | 0 | | | 2 | | | 2 | | | 0 | | | 1 | | | 609 | | | | 2000 | | | | 2000 | | | | 2000 | | | | 607 | | | | 611 | | | |  |
| **P1B/C.21** | | 3 | | | 0 | | | 12 | | | 0 | | | 0 | | | 3 | | | 0 | | | 0 | | | 3 | | | 1 | | | 0 | | | 2 | | | 1 | | | 0 | | | 2 | | | 1 | | | 0 | | | 2 | | | 712 | | | | 2000 | | | | 2000 | | | | 517 | | | | 706 | | | | 913 | | | |  |
| **P1B/C.22** | | 10 | | | 1 | | | 4 | | | 2 | | | 0 | | | 1 | | | 2 | | | 0 | | | 1 | | | 2 | | | 0 | | | 1 | | | 2 | | | 1 | | | 0 | | | 2 | | | 0 | | | 1 | | | 543 | | | | 335 | | | | 534 | | | | 541 | | | | 641 | | | | 664 | | | |  |
| **P1B/C.23** | | 15 | | | 0 | | | 0 | | | 3 | | | 0 | | | 0 | | | 3 | | | 0 | | | 0 | | | 3 | | | 0 | | | 0 | | | 3 | | | 0 | | | 0 | | | 3 | | | 0 | | | 0 | | | 825 | | | | 864 | | | | 860 | | | | 949 | | | | 719 | | | | 734 | | | |  |
| **P2A** | **Exploration of Memory - Sternberg** | | | | | | | | | | | | | | | | | | | | | | | | | | | | | | | | |  | | |  | | |  | | |  | | |  | | |  | | |  | | |  | | |  | | | |  | | | |  | | | |  | | | |  | | | |  | | |
| Patient 2A | TRA | | | TWA | | | TAA | | | RA2 | | | WA2 | | | AA2 | | | RA3 | | | WA3 | | | AA3 | | | RA4 | | | WA4 | | | AA4 | | | RA5 | | | WA5 | | | AA5 | | | RA6 | | | WA6 | | | AA6 | | | TTA | | | TA2 | | | | TA3 | | | | TA4 | | | | TA5 | | | | TA6 | | | |  | | |
| **P2A.1** | 5 | | | 0 | | | 10 | | | 2 | | | 0 | | | 1 | | | 1 | | | 0 | | | 2 | | | 1 | | | 0 | | | 2 | | | 1 | | | 0 | | | 2 | | | 0 | | | 0 | | | 3 | | | 618 | | | 697 | | | | 520 | | | | 615 | | | | 562 | | | | 2000 | | | |  | | |
| **P2A.2** | 11 | | | 2 | | | 2 | | | 3 | | | 0 | | | 0 | | | 3 | | | 0 | | | 0 | | | 2 | | | 0 | | | 1 | | | 1 | | | 1 | | | 1 | | | 2 | | | 1 | | | 0 | | | 671 | | | 762 | | | | 665 | | | | 623 | | | | 540 | | | | 657 | | | |  | | |
| **P2A.3** | 14 | | | 1 | | | 0 | | | 3 | | | 0 | | | 0 | | | 3 | | | 0 | | | 0 | | | 3 | | | 0 | | | 0 | | | 3 | | | 0 | | | 0 | | | 2 | | | 1 | | | 0 | | | 722 | | | 693 | | | | 597 | | | | 796 | | | | 738 | | | | 820 | | | |  | | |
| **P2A.4** | 12 | | | 3 | | | 0 | | | 3 | | | 0 | | | 0 | | | 3 | | | 0 | | | 0 | | | 2 | | | 1 | | | 0 | | | 2 | | | 1 | | | 0 | | | 2 | | | 1 | | | 0 | | | 754 | | | 593 | | | | 678 | | | | 948 | | | | 862 | | | | 809 | | | |  | | |
| **P2A.5** | 9 | | | 5 | | | 1 | | | 2 | | | 1 | | | 0 | | | 2 | | | 1 | | | 0 | | | 1 | | | 1 | | | 1 | | | 2 | | | 1 | | | 0 | | | 2 | | | 1 | | | 0 | | | 596 | | | 630 | | | | 624 | | | | 643 | | | | 669 | | | | 435 | | | |  | | |
| **P2B/C** | | **Exploration of Memory - Sternberg** | | | | | | | | | | | | | | | | | | | | | | | | | | | | | | | | |  | | |  | | |  | | |  | | |  | | |  | | |  | | |  | | | |  | | | |  | | | |  | | | |  | | | |  | | | |  |
| Patient 2B/C | | TRA | | | TWA | | | TAA | | | RA2 | | | WA2 | | | AA2 | | | RA3 | | | WA3 | | | AA3 | | | RA4 | | | WA4 | | | AA4 | | | RA5 | | | WA5 | | | AA5 | | | RA6 | | | WA6 | | | AA6 | | | TTA | | | | TA2 | | | | TA3 | | | | TA4 | | | | TA5 | | | | TA6 | | | |  |
| **P2B/C.1** | | 10 | | | 0 | | | 5 | | | 2 | | | 0 | | | 1 | | | 2 | | | 0 | | | 1 | | | 2 | | | 0 | | | 1 | | | 2 | | | 0 | | | 1 | | | 2 | | | 0 | | | 1 | | | 618 | | | | 532 | | | | 808 | | | | 553 | | | | 610 | | | | 587 | | | |  |
| **P2B/C.2** | | 7 | | | 7 | | | 1 | | | 1 | | | 2 | | | 0 | | | 2 | | | 1 | | | 0 | | | 2 | | | 1 | | | 0 | | | 2 | | | 1 | | | 0 | | | 0 | | | 2 | | | 1 | | | 796 | | | | 742 | | | | 766 | | | | 811 | | | | 836 | | | | 2000 | | | |  |
| **P2B/C.3** | | 11 | | | 3 | | | 1 | | | 3 | | | 0 | | | 0 | | | 2 | | | 1 | | | 0 | | | 2 | | | 1 | | | 0 | | | 3 | | | 0 | | | 0 | | | 1 | | | 1 | | | 1 | | | 849 | | | | 670 | | | | 1184 | | | | 1020 | | | | 723 | | | | 755 | | | |  |
| **P2B/C.4** | | 10 | | | 5 | | | 0 | | | 2 | | | 1 | | | 0 | | | 2 | | | 1 | | | 0 | | | 2 | | | 1 | | | 0 | | | 2 | | | 1 | | | 0 | | | 2 | | | 1 | | | 0 | | | 475 | | | | 410 | | | | 600 | | | | 491 | | | | 422 | | | | 452 | | | |  |
| **P2B/C.5** | | 3 | | | 2 | | | 10 | | | 1 | | | 0 | | | 2 | | | 0 | | | 0 | | | 3 | | | 0 | | | 1 | | | 2 | | | 1 | | | 1 | | | 1 | | | 1 | | | 0 | | | 2 | | | 399 | | | | 636 | | | | 2000 | | | | 2000 | | | | 502 | | | | 332 | | | |  |
| **P2B/C.6** | | 9 | | | 1 | | | 5 | | | 2 | | | 0 | | | 1 | | | 1 | | | 0 | | | 2 | | | 2 | | | 0 | | | 1 | | | 2 | | | 0 | | | 1 | | | 2 | | | 1 | | | 0 | | | 992 | | | | 1003 | | | | 836 | | | | 1160 | | | | 796 | | | | 1086 | | | |  |
| **P2B/C.8** | | 8 | | | 2 | | | 5 | | | 1 | | | 0 | | | 2 | | | 1 | | | 0 | | | 2 | | | 2 | | | 1 | | | 0 | | | 2 | | | 0 | | | 1 | | | 2 | | | 1 | | | 0 | | | 668 | | | | 493 | | | | 823 | | | | 632 | | | | 680 | | | | 703 | | | |  |
| **P2B/C.10** | | 8 | | | 4 | | | 3 | | | 2 | | | 0 | | | 1 | | | 2 | | | 1 | | | 0 | | | 0 | | | 1 | | | 2 | | | 2 | | | 1 | | | 0 | | | 2 | | | 1 | | | 0 | | | 466 | | | | 532 | | | | 547 | | | | 2000 | | | | 384 | | | | 400 | | | |  |
| **P2B/C.11** | | 15 | | | 0 | | | 0 | | | 3 | | | 0 | | | 0 | | | 3 | | | 0 | | | 0 | | | 3 | | | 0 | | | 0 | | | 3 | | | 0 | | | 0 | | | 3 | | | 0 | | | 0 | | | 796 | | | | 725 | | | | 659 | | | | 742 | | | | 934 | | | | 918 | | | |  |
| **P2B/C.12** | | 7 | | | 1 | | | 7 | | | 2 | | | 0 | | | 1 | | | 2 | | | 0 | | | 1 | | | 0 | | | 0 | | | 3 | | | 2 | | | 0 | | | 1 | | | 1 | | | 1 | | | 1 | | | 1161 | | | | 1480 | | | | 950 | | | | 2000 | | | | 927 | | | | 1411 | | | |  |
| **P2B/C.13** | | 7 | | | 0 | | | 8 | | | 1 | | | 0 | | | 2 | | | 1 | | | 0 | | | 2 | | | 2 | | | 0 | | | 1 | | | 2 | | | 0 | | | 1 | | | 1 | | | 0 | | | 2 | | | 634 | | | | 422 | | | | 535 | | | | 678 | | | | 831 | | | | 464 | | | |  |
| **P2B/C.14** | | 7 | | | 1 | | | 7 | | | 0 | | | 1 | | | 2 | | | 1 | | | 0 | | | 2 | | | 2 | | | 0 | | | 1 | | | 2 | | | 0 | | | 1 | | | 2 | | | 0 | | | 1 | | | 553 | | | | 2000 | | | | 484 | | | | 499 | | | | 545 | | | | 650 | | | |  |
| **P2B/C.15** | | 7 | | | 4 | | | 4 | | | 1 | | | 1 | | | 1 | | | 1 | | | 1 | | | 1 | | | 2 | | | 1 | | | 0 | | | 2 | | | 1 | | | 0 | | | 1 | | | 0 | | | 2 | | | 916 | | | | 745 | | | | 302 | | | | 580 | | | | 1659 | | | | 888 | | | |  |
| **P2B/C.16** | | 10 | | | 0 | | | 5 | | | 2 | | | 0 | | | 1 | | | 2 | | | 0 | | | 1 | | | 2 | | | 0 | | | 1 | | | 2 | | | 0 | | | 1 | | | 2 | | | 0 | | | 1 | | | 568 | | | | 511 | | | | 506 | | | | 625 | | | | 526 | | | | 669 | | | |  |
| **P2B/C.18** | | 6 | | | 3 | | | 6 | | | 1 | | | 0 | | | 2 | | | 1 | | | 1 | | | 1 | | | 2 | | | 1 | | | 0 | | | 1 | | | 0 | | | 2 | | | 1 | | | 1 | | | 1 | | | 549 | | | | 1381 | | | | 255 | | | | 552 | | | | 293 | | | | 261 | | | |  |
| **P2B/C.19** | | 8 | | | 3 | | | 4 | | | 2 | | | 0 | | | 1 | | | 2 | | | 0 | | | 1 | | | 1 | | | 1 | | | 1 | | | 2 | | | 0 | | | 1 | | | 1 | | | 2 | | | 0 | | | 661 | | | | 533 | | | | 720 | | | | 686 | | | | 544 | | | | 1011 | | | |  |
| **P2B/C.23** | | 7 | | | 4 | | | 4 | | | 1 | | | 2 | | | 0 | | | 1 | | | 1 | | | 1 | | | 2 | | | 1 | | | 0 | | | 1 | | | 0 | | | 2 | | | 2 | | | 0 | | | 1 | | | 811 | | | | 870 | | | | 821 | | | | 922 | | | | 636 | | | | 752 | | | |  |
| **P2B/C.24** | | 7 | | | 5 | | | 3 | | | 1 | | | 1 | | | 1 | | | 1 | | | 1 | | | 1 | | | 1 | | | 1 | | | 1 | | | 2 | | | 1 | | | 0 | | | 2 | | | 1 | | | 0 | | | 673 | | | | 1537 | | | | 845 | | | | 299 | | | | 535 | | | | 478 | | | |  |
| **Control** | | | **Exploration of Memory - Sternberg** | | | | | | | | | | | | | | | | | | | | | | | | | | | | | | | | |  | | |  | | |  | | |  | | |  | | |  | | |  | | |  | | | |  | | | |  | | | |  | | | |  | | | |  | | | |
| VIH(-) | | | TRA | | | TWA | | | TAA | | | RA2 | | | WA2 | | | AA2 | | | RA3 | | | WA3 | | | AA3 | | | RA4 | | | WA4 | | | AA4 | | | RA5 | | | WA5 | | | AA5 | | | RA6 | | | WA6 | | | AA6 | | | TTA | | | | TA2 | | | | TA3 | | | | TA4 | | | | TA5 | | | | TA6 | | | |
| CMem.1 | | | 12 | | | 3 | | | 0 | | | 3 | | | 0 | | | 0 | | | 2 | | | 1 | | | 0 | | | 2 | | | 1 | | | 0 | | | 2 | | | 1 | | | 0 | | | 3 | | | 0 | | | 0 | | | 531 | | | | 384 | | | | 428 | | | | 378 | | | | 802 | | | | 668 | | | |
| CMem.2 | | | 13 | | | 2 | | | 0 | | | 3 | | | 0 | | | 0 | | | 2 | | | 1 | | | 0 | | | 2 | | | 1 | | | 0 | | | 3 | | | 0 | | | 0 | | | 3 | | | 0 | | | 0 | | | 666 | | | | 576 | | | | 723 | | | | 765 | | | | 620 | | | | 699 | | | |
| CMem.3 | | | 14 | | | 1 | | | 0 | | | 3 | | | 0 | | | 0 | | | 3 | | | 0 | | | 0 | | | 3 | | | 0 | | | 0 | | | 2 | | | 1 | | | 0 | | | 3 | | | 0 | | | 0 | | | 885 | | | | 861 | | | | 769 | | | | 709 | | | | 1189 | | | | 998 | | | |
| CMem.4 | | | 14 | | | 1 | | | 0 | | | 3 | | | 0 | | | 0 | | | 3 | | | 0 | | | 0 | | | 3 | | | 0 | | | 0 | | | 3 | | | 0 | | | 0 | | | 2 | | | 1 | | | 0 | | | 507 | | | | 453 | | | | 485 | | | | 470 | | | | 580 | | | | 572 | | | |
| CMem.5 | | | 14 | | | 1 | | | 0 | | | 2 | | | 1 | | | 0 | | | 3 | | | 0 | | | 0 | | | 3 | | | 0 | | | 0 | | | 3 | | | 0 | | | 0 | | | 3 | | | 0 | | | 0 | | | 504 | | | | 385 | | | | 455 | | | | 533 | | | | 614 | | | | 495 | | | |
| CMem.6 | | | 12 | | | 3 | | | 0 | | | 3 | | | 0 | | | 0 | | | 3 | | | 0 | | | 0 | | | 2 | | | 1 | | | 0 | | | 2 | | | 1 | | | 0 | | | 2 | | | 1 | | | 0 | | | 525 | | | | 478 | | | | 545 | | | | 602 | | | | 593 | | | | 420 | | | |
| CMem.7 | | | 12 | | | 3 | | | 0 | | | 2 | | | 1 | | | 0 | | | 3 | | | 0 | | | 0 | | | 3 | | | 0 | | | 0 | | | 2 | | | 1 | | | 0 | | | 2 | | | 1 | | | 0 | | | 916 | | | | 1018 | | | | 753 | | | | 1024 | | | | 886 | | | | 931 | | | |
| CMem.8 | | | 15 | | | 0 | | | 0 | | | 3 | | | 0 | | | 0 | | | 3 | | | 0 | | | 0 | | | 3 | | | 0 | | | 0 | | | 3 | | | 0 | | | 0 | | | 3 | | | 0 | | | 0 | | | 901 | | | | 721 | | | | 712 | | | | 781 | | | | 1179 | | | | 1111 | | | |
| CMem.9 | | | 13 | | | 2 | | | 0 | | | 3 | | | 0 | | | 0 | | | 3 | | | 0 | | | 0 | | | 3 | | | 0 | | | 0 | | | 3 | | | 0 | | | 0 | | | 1 | | | 2 | | | 0 | | | 891 | | | | 838 | | | | 819 | | | | 940 | | | | 886 | | | | 1135 | | | |
| CMem.10 | | | 13 | | | 2 | | | 0 | | | 3 | | | 0 | | | 0 | | | 3 | | | 0 | | | 0 | | | 2 | | | 1 | | | 0 | | | 2 | | | 1 | | | 0 | | | 3 | | | 0 | | | 0 | | | 781 | | | | 765 | | | | 752 | | | | 842 | | | | 970 | | | | 659 | | | |
| CMem.11 | | | 15 | | | 0 | | | 0 | | | 3 | | | 0 | | | 0 | | | 3 | | | 0 | | | 0 | | | 3 | | | 0 | | | 0 | | | 3 | | | 0 | | | 0 | | | 3 | | | 0 | | | 0 | | | 608 | | | | 554 | | | | 566 | | | | 555 | | | | 694 | | | | 669 | | | |
| CMem.12 | | | 13 | | | 0 | | | 2 | | | 3 | | | 0 | | | 0 | | | 3 | | | 0 | | | 0 | | | 3 | | | 0 | | | 0 | | | 2 | | | 0 | | | 1 | | | 2 | | | 0 | | | 1 | | | 792 | | | | 883 | | | | 691 | | | | 654 | | | | 923 | | | | 883 | | | |
| CMem.13 | | | 13 | | | 1 | | | 1 | | | 3 | | | 0 | | | 0 | | | 3 | | | 0 | | | 0 | | | 2 | | | 1 | | | 0 | | | 3 | | | 0 | | | 0 | | | 2 | | | 0 | | | 1 | | | 704 | | | | 672 | | | | 759 | | | | 580 | | | | 717 | | | | 776 | | | |
| CMem.14 | | | 14 | | | 1 | | | 0 | | | 3 | | | 0 | | | 0 | | | 3 | | | 0 | | | 0 | | | 3 | | | 0 | | | 0 | | | 3 | | | 0 | | | 0 | | | 2 | | | 1 | | | 0 | | | 1044 | | | | 1000 | | | | 995 | | | | 1106 | | | | 993 | | | | 1165 | | | |
| CMem.15 | | | 14 | | | 1 | | | 0 | | | 3 | | | 0 | | | 0 | | | 2 | | | 1 | | | 0 | | | 3 | | | 0 | | | 0 | | | 3 | | | 0 | | | 0 | | | 3 | | | 0 | | | 0 | | | 654 | | | | 564 | | | | 627 | | | | 625 | | | | 638 | | | | 806 | | | |
| CMem.16 | | | 8 | | | 7 | | | 0 | | | 0 | | | 3 | | | 0 | | | 3 | | | 0 | | | 0 | | | 1 | | | 2 | | | 0 | | | 2 | | | 1 | | | 0 | | | 2 | | | 1 | | | 0 | | | 538 | | | | 2000 | | | | 483 | | | | 573 | | | | 605 | | | | 537 | | | |
| CMem.17 | | | 10 | | | 4 | | | 1 | | | 3 | | | 0 | | | 0 | | | 2 | | | 1 | | | 0 | | | 2 | | | 1 | | | 0 | | | 1 | | | 1 | | | 1 | | | 2 | | | 1 | | | 0 | | | 651 | | | | 745 | | | | 652 | | | | 684 | | | | 403 | | | | 602 | | | |
| CMem.18 | | | 11 | | | 4 | | | 0 | | | 3 | | | 0 | | | 0 | | | 3 | | | 0 | | | 0 | | | 2 | | | 1 | | | 0 | | | 3 | | | 0 | | | 0 | | | 0 | | | 3 | | | 0 | | | 509 | | | | 486 | | | | 498 | | | | 473 | | | | 566 | | | | 2000 | | | |
| CMem.19 | | | 14 | | | 1 | | | 0 | | | 3 | | | 0 | | | 0 | | | 3 | | | 0 | | | 0 | | | 3 | | | 0 | | | 0 | | | 3 | | | 0 | | | 0 | | | 2 | | | 1 | | | 0 | | | 542 | | | | 465 | | | | 458 | | | | 543 | | | | 667 | | | | 595 | | | |
| CMem.20 | | | 14 | | | 1 | | | 0 | | | 3 | | | 0 | | | 0 | | | 3 | | | 0 | | | 0 | | | 2 | | | 1 | | | 0 | | | 3 | | | 0 | | | 0 | | | 3 | | | 0 | | | 0 | | | 701 | | | | 518 | | | | 648 | | | | 578 | | | | 1073 | | | | 647 | | | |
| CMem.21 | | | 14 | | | 1 | | | 0 | | | 3 | | | 0 | | | 0 | | | 3 | | | 0 | | | 0 | | | 3 | | | 0 | | | 0 | | | 3 | | | 0 | | | 0 | | | 2 | | | 1 | | | 0 | | | 684 | | | | 668 | | | | 581 | | | | 700 | | | | 795 | | | | 674 | | | |
| CMem.22 | | | 13 | | | 2 | | | 0 | | | 3 | | | 0 | | | 0 | | | 3 | | | 0 | | | 0 | | | 1 | | | 2 | | | 0 | | | 3 | | | 0 | | | 0 | | | 3 | | | 0 | | | 0 | | | 504 | | | | 418 | | | | 458 | | | | 425 | | | | 576 | | | | 591 | | | |
| CMem.23 | | | 15 | | | 0 | | | 0 | | | 3 | | | 0 | | | 0 | | | 3 | | | 0 | | | 0 | | | 3 | | | 0 | | | 0 | | | 3 | | | 0 | | | 0 | | | 3 | | | 0 | | | 0 | | | 531 | | | | 558 | | | | 470 | | | | 477 | | | | 545 | | | | 606 | | | |
| CMem.24 | | | 15 | | | 0 | | | 0 | | | 3 | | | 0 | | | 0 | | | 3 | | | 0 | | | 0 | | | 3 | | | 0 | | | 0 | | | 3 | | | 0 | | | 0 | | | 3 | | | 0 | | | 0 | | | 570 | | | | 519 | | | | 528 | | | | 553 | | | | 606 | | | | 645 | | | |
| CMem.25 | | | 12 | | | 3 | | | 0 | | | 2 | | | 1 | | | 0 | | | 3 | | | 0 | | | 0 | | | 2 | | | 1 | | | 0 | | | 2 | | | 1 | | | 0 | | | 3 | | | 0 | | | 0 | | | 550 | | | | 473 | | | | 558 | | | | 465 | | | | 52 | | | | 666 | | | |
| CMem.26 | | | 13 | | | 2 | | | 0 | | | 3 | | | 0 | | | 0 | | | 2 | | | 1 | | | 0 | | | 3 | | | 0 | | | 0 | | | 3 | | | 0 | | | 0 | | | 2 | | | 1 | | | 0 | | | 571 | | | | 479 | | | | 735 | | | | 522 | | | | 612 | | | | 556 | | | |
| CMem.27 | | | 14 | | | 1 | | | 0 | | | 3 | | | 0 | | | 0 | | | 3 | | | 0 | | | 0 | | | 3 | | | 0 | | | 0 | | | 2 | | | 1 | | | 0 | | | 3 | | | 0 | | | 0 | | | 874 | | | | 704 | | | | 959 | | | | 754 | | | | 1125 | | | | 911 | | | |
| CMem.28 | | | 15 | | | 0 | | | 0 | | | 3 | | | 0 | | | 0 | | | 3 | | | 0 | | | 0 | | | 3 | | | 0 | | | 0 | | | 3 | | | 0 | | | 0 | | | 3 | | | 0 | | | 0 | | | 864 | | | | 772 | | | | 799 | | | | 842 | | | | 821 | | | | 1085 | | | |
| CMem.29 | | | 14 | | | 1 | | | 0 | | | 3 | | | 0 | | | 0 | | | 3 | | | 0 | | | 0 | | | 2 | | | 1 | | | 0 | | | 3 | | | 0 | | | 0 | | | 3 | | | 0 | | | 0 | | | 697 | | | | 680 | | | | 680 | | | | 632 | | | | 818 | | | | 655 | | | |
| CMem.30 | | | 14 | | | 1 | | | 0 | | | 3 | | | 0 | | | 0 | | | 3 | | | 0 | | | 0 | | | 3 | | | 0 | | | 0 | | | 2 | | | 1 | | | 0 | | | 3 | | | 0 | | | 0 | | | 791 | | | | 521 | | | | 661 | | | | 986 | | | | 704 | | | | 1052 | | | |
| CMem.31 | | | 14 | | | 1 | | | 0 | | | 3 | | | 0 | | | 0 | | | 3 | | | 0 | | | 0 | | | 2 | | | 1 | | | 0 | | | 3 | | | 0 | | | 0 | | | 3 | | | 0 | | | 0 | | | 740 | | | | 616 | | | | 649 | | | | 760 | | | | 757 | | | | 925 | | | |
| CMem.32 | | | 14 | | | 1 | | | 0 | | | 3 | | | 0 | | | 0 | | | 3 | | | 0 | | | 0 | | | 3 | | | 0 | | | 0 | | | 3 | | | 0 | | | 0 | | | 2 | | | 1 | | | 0 | | | 615 | | | | 514 | | | | 724 | | | | 506 | | | | 589 | | | | 806 | | | |
| CMem.33 | | | 15 | | | 0 | | | 0 | | | 3 | | | 0 | | | 0 | | | 3 | | | 0 | | | 0 | | | 3 | | | 0 | | | 0 | | | 3 | | | 0 | | | 0 | | | 3 | | | 0 | | | 0 | | | 597 | | | | 504 | | | | 491 | | | | 524 | | | | 759 | | | | 708 | | | |
| CMem.34 | | | 15 | | | 0 | | | 0 | | | 3 | | | 0 | | | 0 | | | 3 | | | 0 | | | 0 | | | 3 | | | 0 | | | 0 | | | 3 | | | 0 | | | 0 | | | 3 | | | 0 | | | 0 | | | 708 | | | | 541 | | | | 697 | | | | 682 | | | | 922 | | | | 700 | | | |
| CMem.35 | | | 15 | | | 0 | | | 0 | | | 3 | | | 0 | | | 0 | | | 3 | | | 0 | | | 0 | | | 3 | | | 0 | | | 0 | | | 3 | | | 0 | | | 0 | | | 3 | | | 0 | | | 0 | | | 869 | | | | 595 | | | | 719 | | | | 844 | | | | 1065 | | | | 1120 | | | |
| CMem.36 | | | 12 | | | 2 | | | 1 | | | 3 | | | 0 | | | 0 | | | 2 | | | 1 | | | 0 | | | 2 | | | 0 | | | 1 | | | 3 | | | 0 | | | 0 | | | 2 | | | 1 | | | 0 | | | 1085 | | | | 918 | | | | 1165 | | | | 1182 | | | | 1196 | | | | 1032 | | | |
| CMem.37 | | | 12 | | | 3 | | | 0 | | | 3 | | | 0 | | | 0 | | | 3 | | | 0 | | | 0 | | | 2 | | | 1 | | | 0 | | | 3 | | | 0 | | | 0 | | | 1 | | | 2 | | | 0 | | | 874 | | | | 1018 | | | | 522 | | | | 906 | | | | 1075 | | | | 825 | | | |
| CMem.38 | | | 7 | | | 2 | | | 6 | | | 0 | | | 0 | | | 3 | | |  | | |  | | |  | | | 2 | | | 1 | | | 0 | | | 1 | | | 1 | | | 1 | | | 2 | | | 0 | | | 1 | | | 755 | | | | 2000 | | | | 840 | | | | 786 | | | | 699 | | | | 668 | | | |
| CMem.39 | | | 13 | | | 1 | | | 1 | | | 3 | | | 0 | | | 0 | | | 3 | | | 0 | | | 0 | | | 3 | | | 0 | | | 0 | | | 2 | | | 0 | | | 1 | | | 2 | | | 0 | | | 1 | | | 857 | | | | 824 | | | | 839 | | | | 721 | | | | 1194 | | | | 801 | | | |
| CMem.40 | | | 10 | | | 0 | | | 5 | | | 0 | | | 2 | | | 1 | | | 2 | | | 0 | | | 1 | | | 2 | | | 0 | | | 1 | | | 2 | | | 0 | | | 1 | | | 2 | | | 0 | | | 1 | | | 929 | | | | 846 | | | | 673 | | | | 1338 | | | | 841 | | | | 948 | | | |
| CMem.41 | | | 9 | | | 1 | | | 5 | | | 2 | | | 0 | | | 1 | | | 1 | | | 0 | | | 2 | | | 2 | | | 0 | | | 1 | | | 2 | | | 0 | | | 1 | | | 2 | | | 0 | | | 1 | | | 724 | | | | 579 | | | | 783 | | | | 768 | | | | 746 | | | | 774 | | | |

**TRA:** total number of right answers; **TWA:** total number of wrong answers; **TAA:** total number of absence of answers; **RA2-6:** number of right answers in groups from 2 to 6 letters; **WA2-6:** number of wrong answers in groups from 2 to 6 letters; **AA2-6:** number of absence of answers in groups from 2 to 6 letters; **TTA:** mean time to answer (ms); **TA2-6:** time to answer (ms) in groups from 2 to 6 letters.
