# Supplementary material for: Defects in immune response to Toxoplasma gondii are associated with enhanced HIV-1-related neurocognitive impairment in co-infected patients
Source: PLoS One. 2023 May 24;18(5):e0285976. doi: 10.1371/journal.pone.0285976 (PMC10208516; doi:10.1371/journal.pone.0285976)
Supplement: S7 Table — (DOC) [file pone.0285976.s007.doc]

**S7 Table – Wisconsin Card Sorting Test** (WCST)

| **P1A** | **WCST** |  |  |  |
| --- | --- | --- | --- | --- |
| **Patient 1A** | **Categories** | **PE** | **TE** | **FMS** |
| **P1A.1** | 5 | 3 | 24 | 5 |
| **P1A.2** | 1 | 20 | 67 | 7 |
| **P1A.3** | 4 | 12 | 44 | 5 |
| **P1A.4** | 1 | 33 | 82 | 6 |
| **P1A.5** | 6 | 4 | 23 | 1 |
| **P1A.6** | 1 | 21 | 79 | 7 |
| **P1A.7** | 5 | 8 | 32 | 5 |
| **P1A.8** | 6 | 7 | 26 | 2 |
| **P1A.9** | 6 | 10 | 35 | 3 |
| **P1B/C** | **WCST** |  |  |  |
| **Patient 1B/C** | **Categories** | **PE** | **TE** | **FMS** |
| **P1B/C.1** | 6 | 9 | 28 | 0 |
| **P1B/C.2** | 0 | 7 | 78 | 8 |
| **P1B/C.3** | 2 | 6 | 14 | 56 |
| **P1B/C.4** | 0 | 11 | 46 | 12 |
| **P1B/C.5** | 3 | 14 | 41 | 9 |
| **P1B/C.6** | 6 | 8 | 27 | 3 |
| **P1B/C.7** | 4 | 11 | 37 | 6 |
| **P1B/C.8** | 6 | 5 | 5 | 25 |
| **P1B/C.9** | 2 | 41 | 75 | 4 |
| **P1B/C.10** | 4 | 9 | 40 | 5 |
| **P1B/C.11** | 0 | 10 | 99 | 5 |
| **P1B/C.12** | 0 | 0 | 49 | 14 |
| **P1B/C.13** | 1 | 19 | 81 | 6 |
| **P1B/C.14** | 1 | 18 | 60 | 9 |
| **P1B/C.15** | 3 | 6 | 35 | 10 |
| **P1B/C.16** | 1 | 52 | 89 | 4 |
| **P1B/C.17** | 4 | 21 | 51 | 5 |
| **P1B/C.18** | 4 | 7 | 43 | 4 |
| **P1B/C.19** | 6 | 6 | 24 | 1 |
| **P1B/C.20** | 1 | 9 | 79 | 6 |
| **P1B/C.21** | 2 | 6 | 46 | 8 |
| **P1B/C.22** | 3 | 9 | 40 | 9 |
| **P1B/C.23** | 5 | 8 | 30 | 5 |
| **P2A** | **WCST** |  |  |  |
| **Patient 2A** | **Categories** | **PE** | **TE** | **FMS** |
| **P2A.1** | 0 | 6 | 47 | 11 |
| **P2A.2** | 6 | 6 | 28 | 5 |
| **P2A.3** | 4 | 4 | 38 | 6 |
| **P2A.4** | 3 | 9 | 39 | 9 |
| **P2A.5** | 0 | 3 | 91 | 7 |
| **P2B/C** | **WCST** |  |  |  |
| **Patient 2B/C** | **Categories** | **PE** | **TE** | **FMS** |
| **P2B/C.1** | 5 | 14 | 43 | 5 |
| **P2B/C.2** | 6 | 3 | 25 | 4 |
| **P2B/C.3** | 3 | 15 | 55 | 6 |
| **P2B/C.4** | 0 | 5 | 95 | 6 |
| **P2B/C.5** | 1 | 31 | 81 | 6 |
| **P2B/C.6** | 2 | 23 | 72 | 5 |
| **P2B/C.7** | 0 | 7 | 96 | 6 |
| **P2B/C.8** | 1 | 16 | 83 | 5 |
| **P2B/C.9** | 5 | 5 | 43 | 3 |
| **P2B/C.10** | 1 | 11 | 57 | 8 |
| **P2B/C.11** | 6 | 1 | 11 | 2 |
| **P2B/C.12** | 1 | 13 | 51 | 9 |
| **P2B/C.13** | 0 | 1 | 37 | 13 |
| **P2B/C.14** | 3 | 21 | 61 | 5 |
| **P2B/C.15** | 4 | 8 | 46 | 6 |
| **P2B/C.16** | 1 | 38 | 80 | 6 |
| **P2B/C.17** | 0 | 4 | 96 | 6 |
| **P2B/C.18** | 0 | 39 | 98 | 5 |
| **P2B/C.19** | 4 | 7 | 40 | 5 |
| **P2B/C.24** | 0 | 8 | 90 | 7 |
| **Control** | **WCST** |  |  |  |
| **VIH(-)** | **Categories** | **PE** | **TE** | **FMS** |
| CWCS.1 | 6 | 0 | 10 | 1 |
| CWCS.2 | 6 | 3 | 16 | 1 |
| CWCS.3 | 6 | 4 | 24 | 2 |
| CWCS.4 | 6 | 8 | 29 | 3 |
| CWCS.5 | 6 | 2 | 15 | 1 |
| CWCS.6 | 6 | 2 | 13 | 0 |
| CWCS.7 | 6 | 2 | 11 | 0 |
| CWCS.8 | 6 | 1 | 13 | 3 |
| CWCS.9 | 6 | 4 | 17 | 1 |
| CWCS.10 | 6 | 3 | 22 | 3 |
| CWCS.11 | 6 | 1 | 11 | 1 |
| CWCS.12 | 6 | 1 | 15 | 0 |
| CWCS.13 | 6 | 2 | 18 | 4 |
| CWCS.14 | 5 | 11 | 41 | 4 |
| CWCS.15 | 6 | 1 | 11 | 0 |
| CWCS.16 | 6 | 0 | 11 | 0 |
| CWCS.17 | 6 | 0 | 9 | 0 |
| CWCS.18 | 6 | 4 | 23 | 2 |
| CWCS.19 | 6 | 2 | 13 | 0 |

**Categories:** number of categories/series completed; **PE:** number of perseverative errors; **TE:** number of total errors; **FMS:** number of failures to maintain set
