# Supplementary material for: Defects in immune response to Toxoplasma gondii are associated with enhanced HIV-1-related neurocognitive impairment in co-infected patients
Source: PLoS One. 2023 May 24;18(5):e0285976. doi: 10.1371/journal.pone.0285976 (PMC10208516; doi:10.1371/journal.pone.0285976)
Supplement: S8 Table — (DOCX) [file pone.0285976.s008.docx]

**S8 Table. Production of Citokines – Dispersion of values**

| **C1** | **IL-2** | | |  | **IL-10** | | |  | **TNF-α** | | |  | **IFN-γ** | | |
| --- | --- | --- | --- | --- | --- | --- | --- | --- | --- | --- | --- | --- | --- | --- | --- |
|  | **Medium** | **PHA** | **STAg** |  | **Medium** | **PHA** | **SATg** |  | **Medium** | **PHA** | **SATg** |  | **Medium** | **PHA** | **SATg** |
| **Median^a^** | 17 | 23 | 19 |  | 7 | 618 | 13 |  | 3 | 8 | 3 |  | 27 | 52 | 36 |
| **25%^a^** | 16 | 17 | 18 |  | 5 | 420 | 6 |  | 3 | 6 | 2 |  | 22 | 47 | 31 |
| **75%^a^** | 19 | 25 | 41 |  | 9 | 829 | 26 |  | 5 | 58 | 14 |  | 32 | 9491 | 40 |
| **n^b^** | 5 | 5 | 5 |  | 4 | 4 | 4 |  | 5 | 4 | 5 |  | 5 | 5 | 4 |
|  |  |  |  |  |  |  |  |  |  |  |  |  |  |  |  |
| **C2** | **IL-2** | | |  | **IL-10** | | |  | **TNF-α** | | |  | **IFN-γ** | | |
|  | **Medium** | **PHA** | **STAg** |  | **Medium** | **PHA** | **SATg** |  | **Medium** | **PHA** | **SATg** |  | **Medium** | **PHA** | **SATg** |
| **Median** | 18 | 17 | 58 |  | 7 | 914 | 41 |  | 3 | 75 | 82 |  | 35 | 5233 | 37520 |
| **25%** | 16 | 15 | 30 |  | 3 | 763 | 33 |  | 3 | 63 | 51 |  | 28 | 1484 | 13008 |
| **75%** | 19 | 21 | 155 |  | 11 | 1155 | 57 |  | 6 | 408 | 388 |  | 39 | 37819 | 38759 |
| **n** | 9 | 9 | 9 |  | 6 | 7 | 7 |  | 7 | 7 | 7 |  | 6 | 6 | 5 |
|  |  |  |  |  |  |  |  |  |  |  |  |  |  |  |  |
| **P1A** | **IL-2** | | |  | **IL-10** | | |  | **TNF-α** | | |  | **IFN-γ** | | |
|  | **Medium** | **PHA** | **STAg** |  | **Medium** | **PHA** | **SATg** |  | **Medium** | **PHA** | **SATg** |  | **Medium** | **PHA** | **SATg** |
| **Median** | 18 | 18 | 19 |  | 8 | 1196 | 53 |  | 3 | 966 | 5 |  | 32 | 29536 | 42 |
| **25%** | 17 | 17 | 16 |  | 3 | 419 | 39 |  | 2 | 794 | 3 |  | 31 | 15293 | 35 |
| **75%** | 19 | 21 | 20 |  | 16 | 2073 | 68 |  | 3 | 1271 | 14 |  | 36 | 34410 | 50 |
| **n** | 5 | 5 | 5 |  | 4 | 4 | 4 |  | 4 | 4 | 4 |  | 4 | 4 | 4 |
|  |  |  |  |  |  |  |  |  |  |  |  |  |  |  |  |
| **P1B/C** | **IL-2** | | |  | **IL-10** | | |  | **TNF-α** | | |  | **IFN-γ** | | |
|  | **Medium** | **PHA** | **STAg** |  | **Medium** | **PHA** | **SATg** |  | **Medium** | **PHA** | **SATg** |  | **Medium** | **PHA** | **SATg** |
| **Median** | 18 | 19 | 17 |  | 75 | 1244 | 144 |  | 5 | 767 | 7 |  | 35 | 15176 | 40 |
| **25%** | 17 | 18 | 17 |  | 22 | 602 | 20 |  | 4 | 353 | 6 |  | 33 | 7440 | 35 |
| **75%** | 19 | 22 | 18 |  | 129 | 1893 | 167 |  | 13 | 1549 | 8 |  | 52 | 22243 | 45 |
| **n** | 8 | 8 | 7 |  | 7 | 7 | 6 |  | 7 | 7 | 6 |  | 7 | 6 | 6 |
|  |  |  |  |  |  |  |  |  |  |  |  |  |  |  |  |
| **P2A** | **IL-2** | | |  | **IL-10** | | |  | **TNF-α** | | |  | **IFN-γ** | | |
|  | **Medium** | **PHA** | **STAg** |  | **Medium** | **PHA** | **SATg** |  | **Medium** | **PHA** | **SATg** |  | **Medium** | **PHA** | **SATg** |
| **Median** | 16 | 21 | 21 |  | 6 | 791 | 17 |  | 7 | 1053 | 13 |  | 41 | 27952 | 88 |
| **25%** | 13 | 17 | 19 |  | 5 | 335 | 11 |  | 5 | 884 | 6 |  | 32 | 9323 | 43 |
| **75%** | 21 | 24 | 22 |  | 22 | 2353 | 116 |  | 8 | 1274 | 62 |  | 54 | 45960 | 277 |
| **n** | 8 | 8 | 8 |  | 7 | 7 | 7 |  | 6 | 6 | 6 |  | 7 | 7 | 6 |
|  |  |  |  |  |  |  |  |  |  |  |  |  |  |  |  |
| **P2B/C** | **IL-2** | | |  | **IL-10** | | |  | **TNF-α** | | |  | **IFN-γ** | | |
|  | **Medium** | **PHA** | **STAg** |  | **Medium** | **PHA** | **SATg** |  | **Medium** | **PHA** | **SATg** |  | **Medium** | **PHA** | **SATg** |
| **Median** | 18 | 19 | 18 |  | 22 | 490 | 68 |  | 5 | 874 | 76 |  | 37 | 20398 | 65 |
| **25%** | 16 | 13 | 15 |  | 10 | 329 | 27 |  | 3 | 484 | 9 |  | 31 | 11189 | 47 |
| **75%** | 19 | 20 | 21 |  | 39 | 927 | 150 |  | 9 | 1899 | 133 |  | 41 | 33439 | 630 |
| **n** | 13 | 13 | 13 |  | 12 | 12 | 12 |  | 11 | 11 | 11 |  | 12 | 11 | 11 |

**^a^** Values of median, 25 and 75 percentiles are expressed in pg/mL

**^b^** n=number of patients in each group
