# Supplementary material for: Defects in immune response to Toxoplasma gondii are associated with enhanced HIV-1-related neurocognitive impairment in co-infected patients
Source: PLoS One. 2023 May 24;18(5):e0285976. doi: 10.1371/journal.pone.0285976 (PMC10208516; doi:10.1371/journal.pone.0285976)
Supplement: S9 Table — (DOCX) [file pone.0285976.s009.docx]

**S9 Table. Production of cytokines - Statistically significant differences**

|  |  | **IL-2** | |
| --- | --- | --- | --- |
| **Condition** | **Groups** | **vs. Medium** | **vs. C2** |
| **of Cultures** |  | (p-value) | (p-value) |
| **SATg** | **C2** | 0,0011^a^ | - |
|  | **P2A** | ns | 0,0061^a^ |
|  | **P2B/C** | ns | <0,0001^a^ |

|  |  | **IL-10** | | |
| --- | --- | --- | --- | --- |
| **Condition** | **Groups** | **vs. Medium** | **vs. C1** | **vs. P1A** |
| **of Cultures** |  | (p-value) | (p-value) | (p-value) |
| **Medium** | **P1B/C** | - | 0,0424 | 0,0242 |
| **PHA** | **C1** | 0,0286 |  |  |
|  | **P1A** | 0,0286 |  |  |
|  | **P1B/C** | 0,000583 |  |  |
|  | **C2** | 0,00117 |  |  |
|  | **P2A** | 0,000583 |  |  |
|  | **P2B/C** | <0,0001 |  |  |
| **SATg** | **P1A** | 0,0286 | 0,0161^a, b^ |  |
|  | **C2** | 0,00117 | 0,0399^a, b^ |  |
|  | **P2B/C** | 0,00861 |  |  |

|  |  | **TNF-α** | | |
| --- | --- | --- | --- | --- |
| **Condition** | **Groups** | **vs. Medium** | **vs. C1** | **vs. C2** |
| **of Cultures** |  | (p-value) | (p-value) | (p-value) |
| **PHA** | **C1** | 0,0317 |  |  |
|  | **P1A** | 0,0006^a^ | 0,0286 |  |
|  | **P1B/C** | 0,000583 | 0,00606 |  |
|  | **C2** | 0,000583 |  |  |
|  | **P2A** | 0,00216 |  | 0,00117 |
|  | **P2B/C** | <0,0001 |  | 0,0145 |
| **SATg** | **C2** | 0,00117 | 0,00433 |  |
|  | **P2A** | ns |  | 0,0411 |
|  | **P2B/C** | 0,00582 |  |  |

|  |  | **IFN-γ** | | | |
| --- | --- | --- | --- | --- | --- |
| **Condition** | **Groups** | **vs. Medium** | **vs. C1** | **vs. C2** | **vs. P1B/C** |
| **of Cultures** |  | (p-value) | (p-value) | (p-value) | (p-value) |
| **PHA** | **C1** | 0,0159 |  |  |  |
|  | **P1A** | 0,0286 | 0,0158^a, b^ |  |  |
|  | **P1B/C** | 0,00117 |  |  |  |
|  | **C2** | 0,00216 |  |  |  |
|  | **P2A** | 0,00117 |  |  |  |
|  | **P2B/C** | <0,0001 |  |  |  |
| **SATg** | **C2** | 0,0043 | 0,0159 |  |  |
|  | **P2A** | 0,026 |  | 0,0043 |  |
|  | **P2B/C** | 0,0062 |  | 0,00222 | 0,0237 |

Groups were compared using *T- student*^a^ or *Mann-Whitney* tests, as appropriate

"ns" or empty cells: not statistically significant differences

^a^ *T-student* test. All other p-values are for *Mann-Whitney Rank Sum Test*

^b^ The power of the performed test (with alpha=0.0500) is below the desired power of 0.800. Negative finding should be interpreted cautiously.
