# Supplementary material for: Defects in immune response to Toxoplasma gondii are associated with enhanced HIV-1-related neurocognitive impairment in co-infected patients
Source: PLoS One. 2023 May 24;18(5):e0285976. doi: 10.1371/journal.pone.0285976 (PMC10208516; doi:10.1371/journal.pone.0285976)
Supplement: S10 Table — (DOCX) [file pone.0285976.s010.docx]

**S10 Table. Visual P300 Latency - Statistically significant differences**

| **Electrode** | **Group** | **vs. Control^c^** | **vs. P1B/C** |
| --- | --- | --- | --- |
| **Location** |  | (p-value) | (p-value) |
| **Fp1** | **P1A** | 0.0049 |  |
|  | **P1B/C** | 0.0042 |  |
|  | **P2B/C** | 0.0010 |  |
| **Fp2** | **P1A** | 0.0041 |  |
|  | **P1B/C** | 0.0309^b^ |  |
|  | **P2B/C** | 0.0005 |  |
| **F3** | **P1B/C** | 0.0414^b^ |  |
|  | **P2B/C** | 0.0222^a^ |  |
| **F4** | **P1A** | 0.0367^a^ |  |
| **C3** | **P2B/C** | 0.0404^a^ |  |
| **C4** | **P1A** | 0.0304^b^ |  |
|  | **P2B/C** | 0.0196^a^ |  |
| **P3** | **P2B/C** | 0.0124^a^ |  |
| **P4** | **P2B/C** | 0.0120^a^ |  |
| **O1** | **P1B/C** | 0.0359 |  |
|  | **P2B/C** | 0.0334^b^ |  |
| **O2** | **P1A** | 0.0182^a^ |  |
|  | **P1B/C** | 0.0426^b^ |  |
|  | **P2B/C** | <0.0001^a^ | 0.0172^b^ |
| **F8** | **P2B/C** | 0.0188^b^ |  |
| **T6** | **P1A** | 0.0274^a^ |  |
|  | **P1B/C** | 0.0479^b^ |  |
|  | **P2B/C** | 0.0002 |  |
| **Fz** | **P1A** | 0.0201^b^ |  |
|  | **P2B/C** | 0.0046^a^ |  |
| **Cz** | **P1A** | 0.0249^b^ |  |
|  | **P2B/C** | 0.0088^a^ |  |
| **Pz** | **P2B/C** | 0.0037^a^ |  |
| **Oz** | **P1B/C** | 0.0431^a^ |  |
|  | **P2B/C** | 0.0015^a^ | 0.0268^b^ |

Groups were compared using *T- student* or *Mann-Whitney*^a^ tests, as appropriate

Empty cells: not statistically significant differences

^a^ *Mann-Whitney Rank Sum Test*. All other p-values are for *T-student* test

^b^ The power of the performed test (with alpha=0.0500) is below the desired power of 0.800. Negative finding should be interpreted cautiously.

^c^ Control: Group of HIV-1-non infected individuals
