# Supplementary material for: Defects in immune response to Toxoplasma gondii are associated with enhanced HIV-1-related neurocognitive impairment in co-infected patients
Source: PLoS One. 2023 May 24;18(5):e0285976. doi: 10.1371/journal.pone.0285976 (PMC10208516; doi:10.1371/journal.pone.0285976)
Supplement: S11 Table — (DOCX) [file pone.0285976.s011.docx]

**S11 Table. Auditory P300 Latency - Statistically significant differences**

| **Electrode** | **Group** | **vs. Control^c^** |
| --- | --- | --- |
| **Location** |  | (p-value) |
| **Fp1** | **P1A** | 0.0096^a^ |
|  | **P1B/C** | 0.0003 |
| **Fp2** | **P1A** | 0.0045 |
|  | **P1B/C** | <0.0001^a^ |
|  | **P2B/C** | 0.0195^b^ |
| **F3** | **P1B/C** | 0.0035 |
| **F4** | **P1A** | 0.0477^a^ |
|  | **P2B/C** | 0.0062^a^ |
| **C3** | **P1B/C** | <0.0001^a^ |
| **C4** | **P1A** | 0.0295^a^ |
|  | **P1B/C** | <0.0001^a^ |
|  | **P2A** | 0.0243^b^ |
|  | **P2B/C** | 0.0388^a^ |
| **P3** | **P1B/C** | 0.0009 |
|  | **P2B/C** | 0.0351^a^ |
| **P4** | **P1B/C** | 0.0012^a^ |
|  | **P2B/C** | 0.0240^a^ |
| **O1** | **P1B/C** | <0.0001 |
|  | **P2B/C** | 0.0002 |
| **O2** | **P1A** | 0.0026 |
|  | **P1B/C** | <0.0001 |
|  | **P2A** | 0.0492^b^ |
|  | **P2B/C** | 0.0003 |
| **F7** | **P1B/C** | 0.0062^b^ |
| **F8** | **P1A** | 0.0430^a^ |
|  | **P1B/C** | 0.0171^a^ |
| **T3** | **P1B/C** | 0.0030 |
|  | **P2B/C** | 0.0194^a^ |
| **T4** | **P1A** | 0.0363^b^ |
|  | **P1B/C** | 0.0020^a^ |
|  | **P2B/C** | 0.0363^a^ |
| **T5** | **P1B/C** | 0.0014 |
|  | **P2B/C** | 0.0060^b^ |
| **T6** | **P1A** | 0.0477^a^ |
|  | **P1B/C** | <0.0001 |
|  | **P2B/C** | 0.0122^a^ |
| **Fz** | **P1A** | 0.0430^a^ |
|  | **P1B/C** | 0.0003 |
| **Cz** | **P1B/C** | <0.0001^a^ |
|  | **P2B/C** | 0.0442^a^ |
| **Pz** | **P1B/C** | 0.0002 |
|  | **P2A** | 0.0250^b^ |
|  | **P2B/C** | 0.0018 |
| **Oz** | **P1B/C** | <0.0001^a^ |
|  | **P2A** | 0.0472^a^ |
|  | **P2B/C** | 0.0014^a^ |

Groups were compared using *T- student* or *Mann-Whitney*^a^ tests, as appropriate

^a^ *Mann-Whitney Rank Sum Test*. All other p-values are for *T-student* test

^b^ The power of the performed test (with alpha=0.0500) is below the desired power of 0.800. Negative finding should be interpreted cautiously.

^c^ Control: Group of HIV-1-non infected individuals
