# Supplementary material for: Defects in immune response to Toxoplasma gondii are associated with enhanced HIV-1-related neurocognitive impairment in co-infected patients
Source: PLoS One. 2023 May 24;18(5):e0285976. doi: 10.1371/journal.pone.0285976 (PMC10208516; doi:10.1371/journal.pone.0285976)
Supplement: S12 Table — (DOCX) [file pone.0285976.s012.docx]

**S12 Table. Auditory P300 Amplitude - Statistically significant differences**

| **Electrode** | **Group** | **vs. Control^c^** | **vs. P1A** |
| --- | --- | --- | --- |
| **Location** |  | (p-value) | (p-value) |
| **Fp1** | **P2A** |  | 0.0455^a^ |
| **F4** | **P1A** | 0.0493^b^ |  |
|  | **P1B/C** | 0.0177^a^ |  |
|  | **P2A** | 0.0447^b^ |  |
| **Fz** | **P1B/C** | 0.0393^b^ |  |
|  | **P2A** | 0.0392^b^ |  |

Groups were compared using *T- student* or *Mann-Whitney*^a^ tests, as appropriate

Empty cells: not statistically significant differences

^a^ *Mann-Whitney Rank Sum Test*. All other p-values are for *T-student* test

^b^ The power of the performed test (with alpha=0.0500) is below the desired power of 0.800. Negative finding should be interpreted cautiously.

^c^ Control: Group of HIV-1-non infected individuals
