# Supplementary material for: Defects in immune response to Toxoplasma gondii are associated with enhanced HIV-1-related neurocognitive impairment in co-infected patients
Source: PLoS One. 2023 May 24;18(5):e0285976. doi: 10.1371/journal.pone.0285976 (PMC10208516; doi:10.1371/journal.pone.0285976)
Supplement: S13 Table — (DOCX) [file pone.0285976.s013.docx]

**S13 Table. Exploration of Memory - Statistically significant differences**

| **Answers** | **Group** | **vs. Control^a^** | **vs. P1A** |
| --- | --- | --- | --- |
|  |  | (p-value) | (p-value) |
| **Right Answers** | **P1A** | 0.0100 |  |
|  | **P1B/C** | <0.0001 |  |
|  | **P2A** | 0.0389 |  |
|  | **P2B/C** | <0.0001 |  |
| **Wrong Answers** | **P2A** | ns | 0.0433 |
| **(Sets of 6 letters)** |  |  |  |
| **Abcense of Answers** | **P1A** | 0.0013 |  |
|  | **P1B/C** | <0.0001 |  |
|  | **P2B/C** | <0.0001 |  |

Groups were compared using *Mann-Whitney rank sum test*, as appropriate

"ns" or empty cells: not statistically significant differences

^a^ Control: Group of HIV-1-non infected individuals
