# Supplementary material for: Defects in immune response to Toxoplasma gondii are associated with enhanced HIV-1-related neurocognitive impairment in co-infected patients
Source: PLoS One. 2023 May 24;18(5):e0285976. doi: 10.1371/journal.pone.0285976 (PMC10208516; doi:10.1371/journal.pone.0285976)
Supplement: S14 Table — (DOCX) [file pone.0285976.s014.docx]

**S14 Table. Wisconsin Card Sorting Test - Statistically significant differences**

| **Parameter** | **Group** | **vs. Control^c^** | **vs. P1A** |
| --- | --- | --- | --- |
|  |  | (p-value) | (p-value) |
| **Categories Completed** | **P1A** | 0.0075 |  |
|  | **P1B/C** | <0.0001 |  |
|  | **P2A** | 0.0079 |  |
|  | **P2B/C** | <0.0001 |  |
| **Perseverative Errors** | **P1A** | <0.0001 |  |
|  | **P1B/C** | <0.0001 |  |
|  | **P2A** | 0.0208 |  |
|  | **P2B/C** | <0.0001 |  |
| **Total Errors** | **P1A** | <0.0001 |  |
|  | **P1B/C** | <0.0001 |  |
|  | **P2A** | 0.0022 |  |
|  | **P2B/C** | <0.0001 |  |
| **Failures to Maintain Set** | **P1A** | <0.0001^a^ |  |
|  | **P1B/C** | <0.0001 |  |
|  | **P2A** | <0.0001 | 0.0304^a, b^ |
|  | **P2B/C** | <0.0001 |  |

Groups were compared using *T- student*^a^ or *Mann-Whitney* tests, as appropriate

Empty cells: not statistically significant differences

^a^ *T-student* test. All other p-values are for *Mann-Whitney Rank Sum Test*

^b^ The power of the performed test (with alpha=0.0500) is below the desired power of 0.800. Negative finding should be interpreted cautiously.

^c^ Control: Group of HIV-1-non infected individuals
