# Supplementary material for: Defects in immune response to Toxoplasma gondii are associated with enhanced HIV-1-related neurocognitive impairment in co-infected patients
Source: PLoS One. 2023 May 24;18(5):e0285976. doi: 10.1371/journal.pone.0285976 (PMC10208516; doi:10.1371/journal.pone.0285976)
Supplement: Attachment — Submitted filename: PONE-D-22-08134.docx [file pone.0285976.s015.docx]

**In abstract session:**

- For expression significantly, should be mention p-value.
- Statistical tests and software used for data analysis are mentioned.

**In introduction session:**

- The aim of the study should be clearly explain.

**In methods session:**

- Mention the study time.
- Mention the software used to analyze the data and its version.
- Mention the ethics code.

**In results session:**

- In some diagrams, did not mention the horizontal axis title.
- Abbreviations (C1, P1A, etc.) should be written in full below the image.
