# Supplementary material for: Defects in immune response to Toxoplasma gondii are associated with enhanced HIV-1-related neurocognitive impairment in co-infected patients
Source: PLoS One. 2023 May 24;18(5):e0285976. doi: 10.1371/journal.pone.0285976 (PMC10208516; doi:10.1371/journal.pone.0285976)
Supplement: Attachment — Submitted filename: Response to Reviewers.docx [file pone.0285976.s016.docx]

**PONE-D-22-08134**

**[EMID:a219c5ac8b2a365f]**

Response to Editor and Reviewers:

1. Please ensure that your manuscript meets PLOS ONE's style requirements, including those for file naming.

**Answer:** We have revised the entire document, trying to meet PLOS ONE's style requirements, including those for file naming.

2. We suggest you thoroughly copyedit your manuscript for language usage, spelling, and grammar. Upon resubmission, please provide the name of the colleague that edited your manuscript.

**Answer:** We were kindly assisted by a colleague in California, who prefers to remain anonymous, to read and proofread the entire manuscript, providing valuable suggestions for improvement.

3. In your Data Availability statement, you have not specified where the minimal data set underlying the results described in your manuscript can be found.

**Answer:** The data set used to reach the conclusions drawn in this manuscript is provided in *S2-7 Tables* (see *Supporting Information*). Two-tailed *T-student* or *Mann-Whitney* tests, as appropriate, were used to compare groups. *Pearson product moment* was used to analyze correlation. *S8 Table* provides medians, 25^th^ and 75^th^ percentiles for cytokines values. *S9-14 Tables* provides all particular p-values. Data in *S2-7 Tables* were used to build graphs.

4. Is the manuscript technically sound, and do the data support the conclusions?
   a. Reviewer #1: Partly

**Answer:** We are providing additional Tables to explain how data were analyzed, and we hope that this additional information could show how data support the conclusions

b. Reviewer #2: Yes

5. Has the statistical analysis been performed appropriately and rigorously?
   a. Reviewer #1: No

**Answer:** We modified the presentation of results, showing p-values for statistically significant results, and provide *S9-14 Tables* with all particular p-values, including information about the method used and the power of performed test. We hope that this additional information could help to explain that statistical analysis been performed appropriately and rigorously.

b. Reviewer #2: N/A

6. Have the authors made all data underlying the findings in their manuscript fully available?
   a. Reviewer #1: Yes
   b. Reviewer #2: Yes
7. Is the manuscript presented in an intelligible fashion and written in Standard English?
   a. Reviewer #1: No

**Answer:** We have thoroughly copyedited our manuscript for language usage, spelling, and grammar. We were kindly assisted by a colleague in California, who read and edited the entire manuscript.

b. Reviewer #2: Yes

8. **Review Comments to the Author**
   a. **Reviewer #1:** The authors aim to investigate to neurocognitive impairment in individuals co-infected with HIV and *Toxoplasma gondii*. The study is straight forward and included the corresponding controls. However, there are problems associated to the organization and presentation of the data. The project would benefit of the following recommendations:
      i. English language needs substantial revision.

**Answer:** As we have mentioned before, we have thoroughly copyedited our manuscript for language usage, spelling, and grammar. A colleague in California read and copyedited the manuscript.

ii. Each graph should present every single subject on each bar graph. Also, the statistical analyses need to be robust since this reviewer is skeptical that several comparisons are significant due to the high variability.

**Answer:** We have modified graph presentation, including every single patient’s value as black dots. We provide every single p-value (*S9-14 Tables*), including information about the method used and the power of the performed test. We hope that this additional information could explain that statistical analysis has been properly performed.

iii. The manuscript needs standard submission organization. For example, each result subsection should state the main findings and each figure needs to include all the graphs for that specific figure (e.g., A, B, C... F). Please see how other papers published in the journal are organized.

**Answer:** We have revised the entire document, trying to meet PLOS ONE's style requirements. We have modified the presentation of results, stating the main findings, showing p-values for statistically significant results and providing all particular p-values (*S9-14 Tables*). Figures presentation has been also modified.

iv. The discussion is really long and an iteration of the results. Therefore, please reduce it, eliminate figure labels in the discussion, summarize each result and compare it to other studies in the area.

**Answer:** We have revised and modified discussion, eliminating figure labels, summarizing results and comparing with other studies. We have tried to reduce it, avoiding iteration, but trying to discuss all the important issues.

b. **Reviewer #2:**
   i. In abstract session:
      1. For expression significantly, should be mention p-value.

**Answer:** *p<0.05* was considered as significant (see *Abstract*, page 3, line 48). We modified the presentation of results, showing p-values for statistically significant results. All particular p-values are provided in *S9-14 Tables*.

2. Statistical tests and software used for data analysis are mentioned.

**Answer:** Groups were compared using *T-student* or *U-Mann-Whitney* tests as appropriate (see *Abstract*, page 3, line 48). Software used to perform statistical analysis of data was SigmaStat® for Windows, Version 1.0 (1992-1994 Jandel Corporation) (see *Statistical analysis*, page 13, lines 240, 241)

ii. In introduction session:
    1. The aim of the study should be clearly explain.

**Answer:** The aim of the study was to evaluate how *T. gondii*-specific response changes in different stages of the HIV-1 infection, and if this change could be associated with modifications in neurocognitive functions. Immune response was assessed by studying cytokine production in response to *T. gondii* antigens, and neurocognitive functions by evaluating auditory and visual P300 cognitive evoked potentials, Sternberg’s speed of memory scanning task and Wisconsin Card Sorting Test (see *Introduction*, pages 5 and 6, lines 91-94).

iii. In methods session:
     1. Mention the study time.

**Answer:** Samples were analyzed and neurophysiological evaluations were performed between May 2007 and December 2009. Manuscript was written between August 2021 and February 2022.

2. Mention the software used to analyze the data and its version.

**Answer:** *SigmaStat*® for Windows, Version 1.0 (1992-1994 Jandel Corporation) was used to perform statistical analysis of data

3. Mention the ethics code.

**Answer:** All participants provided written informed consent and the study was approved by the ethic boards of the participating institutions, in accordance with the *Declaration of Helsinki* (1964-2013) and the *Belmont Report* (1979).

iv. In results session:
    1. In some diagrams, did not mention the horizontal axis title.

**Answer:** Figures presentation has been revised and modified.

2. Abbreviations (C1, P1A, etc.) should be written in full below the image.

**Answer:** Figures legends explain what abbreviations mean: HIV-1-non infected individuals that were seronegative (C1) or seropositive (C2) to *T. gondii*, and HIV-1 infected (P1) or HIV-1/T. gondii co-infected (P2) patients. Patients (P1 and P2) were grouped as early/asymptomatic or late/symptomatic, depending if their peripheral blood CD4+ T lymphocytes counts were higher (P1A and P2A) or lower (P1B/C and P2B/C) than 350/μL, respectively.
