# Supplementary material for: Defects in immune response to Toxoplasma gondii are associated with enhanced HIV-1-related neurocognitive impairment in co-infected patients
Source: PLoS One. 2023 May 24;18(5):e0285976. doi: 10.1371/journal.pone.0285976 (PMC10208516; doi:10.1371/journal.pone.0285976)
Supplement: Attachment — Submitted filename: Response to Reviewers-Feb 2023.docx [file pone.0285976.s017.docx]

**PONE-D-22-08134R1**

**Defects in immune response to Toxoplasma gondii are associated with enhanced HIV-1-related neurocognitive impairment in co-infected patients**

Response to Editor and Reviewers:

**Comments to the Author**

1. If the authors have adequately addressed most of the comments raised in a previous round of review

Reviewer #1: (No Response)

Reviewer #2:

2. **Is the manuscript technically sound, and do the data support the conclusions?**

Reviewer #1: **Partly**

**Answer:** We have revised the entire document, and made deep modifications in results and discussion sections. All data used to reach the conclusions drawn in this manuscript is provided. Tables are also provided to explain how data were analyzed, to show how data support conclusions.

Reviewer #2: **Yes**

3. **Has the statistical analysis been performed appropriately and rigorously?**

Reviewer #1: **Yes**

Reviewer #2: **I Don't Know**

**Answer:** We show p-values for statistically significant results, and provide *S9-14 Tables* with all p-values, including information about the method used and the power of performed test. This information explain that statistical analysis been performed appropriately and rigorously.

4. **Have the authors made all data** underlying the findings in their manuscript fully **available?**

Reviewer #1: **Yes**

Reviewer #2: **Yes**

5. **Is the manuscript presented in an intelligible fashion and written in standard English?**

Reviewer #1: **No**

**Answer:** We kindly asked **Laura Bartoli, MD, from Australia** and **other colleague from California, who prefers to remain anonymous**, to read and proofread the entire manuscript. They provided suggestions for improvement.

Reviewer #2: **Yes**

6. Review Comments to the Author

**Reviewer #1:**

- The authors should **check the format of other PLoS One published papers** and use similar format.
  - **Answer:** We checked other PLoS One published papers to use a similar format in our manuscript.
- Also, the **results' presentation is boring**.
  - **Answer:** we modified results section, to help reader to obtain the information easily.
- The authors should **use the conclusions of each section as title of each result subsection** instead of "Production of IL-10".
  - **Answer:** We modified results section, stating the main findings of each subsection.
- **Figure legends should be in their own subsection at the end of the manuscript** in order instead of being inserted between the result section.
  - **Answer:** we had followed the instructions given to authors in the *Submission Guidelines* document:

**“Figure captions**

Figure captions must be inserted in the text of the manuscript, immediately following the paragraph in which the figure is first cited”. (https://journals.plos.org/plosone/s/submission-guidelines#loc-figures-and-tables

- **The discussion** for the results obtained **should not be longer than 4 pages long**. Presently, the discussion is 8 pages long which is ridiculous. Please be succinct and avoid iteration of the results.
  - **Answer:** we substantially reduced the length of discussion section to meet, as close as possible, the required four pages for discussion.

**Reviewer #2:**

- They need to **include the suggested changes**
  - **Answer:** we had made our best effort to include all suggested changes.
